# Comprehensive landscape of cell death mechanisms: from molecular cross-talk to therapeutic innovation in oncology

**DOI:** 10.3389/fcell.2025.1611055

**Published:** 2025-07-16

**Authors:** Ke Qi, Yongping Mu, Yang Hu, Jiayi Li, Jia Liu

**Affiliations:** ^1^ Inner Mongolia Medical University, Hohhot, Inner Mongolia, China; ^2^ Department of Clinical Laboratory, The First Hospital of Hohhot, Hohhot, Inner Mongolia, China

**Keywords:** cell death, PCD, cancer, mechanism, therapy

## Abstract

Cell death, or programmed cellular termination, represents a fundamental biological phenomenon crucial for maintaining organismal homeostasis. Traditionally conceptualized as a passive terminal state associated with inflammatory responses and elimination of compromised cells, contemporary research has unveiled cell death as a sophisticated regulatory network encompassing diverse modalities, including apoptosis, necrosis, autophagic cell death, and lysosomal cell death, which are classified as programmed cell death, and pyroptosis, necroptosis, and NETosis, which are classified as inflammatory cell death, have been described over the years. Recently, several novel forms of cell death, namely, mitoptosis, paraptosis, immunogenic cell death, entosis, methuosis, parthanatos, ferroptosis, autosis, alkaliptosis, oxeiptosis, cuproptosis, erebosis and disulfidptosis, have been discovered and advanced our understanding of cell death and its complexity. This synthesis examines the historical progression and defining characteristics of cellular termination pathways, with particular emphasis on their molecular regulation and pathophysiological significance. The mechanistic diversity of these processes not only reveals intricate cellular quality control systems but also provides therapeutic opportunities for neoplastic diseases. For instance, investigations into oncogenic regulators like B-cell lymphoma 2 (BCL-2) family proteins have illuminated the critical relationship between apoptotic resistance and malignant progression, catalyzing development of pro-apoptotic agents such as BH3 mimetics. Strategic integration of these targeted therapies with conventional cytotoxic regimens and immunomodulatory approaches represents a promising frontier in precision oncology, potentially enhancing therapeutic efficacy while mitigating adverse effects in cancer management.

## 1 Introduction

Cell death constitutes an essential biological phenomenon marked by sequential functional deterioration leading to terminal cellular breakdown. This mechanism maintains tissue homeostasis by selectively removing malfunctioning and damaged, and potentially detrimental cellular elements (Galluzzi et al., 2018). Cellular termination represents a fundamental biological mechanism characterized by progressive functional impairment culminating in irreversible cellular collapse. This essential process maintains tissue homeostasis through selective clearance of compromised cellular material, disease states, or environmental insults, resulting in detrimental cell loss (Grohmann et al., 2021; Morana et al., 2022; Krysko et al., 2012; Troitskaya et al., 2022).

**FIGURE 1:**
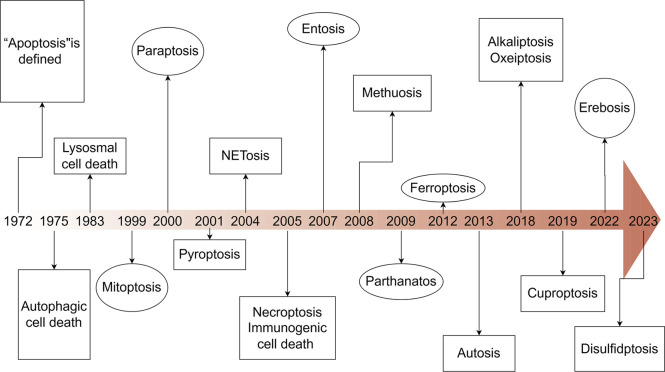
Timeline of the discovery of cell death. This timeline depicts the important discoveries and advancements in cell death research, including the recognition of multiple forms of cell death.

Cell death can be categorized based on specific morphological characteristics, biological context, and triggering mechanisms. In 2018, an international consortium of cell death researchers collaboratively published a seminal article titled “Molecular Mechanisms of Cell Death: Recommendations of the Nomenclature Committee on Cell Death (2018)” in Cell Death and Differentiation. This comprehensive classification system delineates cell death into two primary categories: accidental cell death (ACD) and regulated cell death (RCD). ACD represents an uncontrolled cellular demise mechanism initiated by extreme physical, chemical, or mechanical stress that surpasses cellular regulatory thresholds, inevitably leading to cellular collapse (Galluzzi et al., 2018). RCD is a genetically programmed, autonomous, and tightly controlled cellular mechanism essential for organismal homeostasis. This evolutionarily conserved process is executed through signalosome complex assembly and plays pivotal roles in developmental morphogenesis and immune regulation. Functioning under both physiological and pathological conditions, RCD—also termed programmed cell death (PCD)—eliminates superfluous or compromised cells through molecularly defined pathways (Christgen et al., 2022). The main types of RCDs known today include:apoptosis (Carneiro and El-Deiry, 2020), autophagic cell death (Debnath et al., 2023), lysosomal cell death (Tang et al., 2019), mitoptosis (Maiese, 2024), paraptosis (Xu et al., 2024), pyroptosis (Rao et al., 2022), NETosis (Thiam et al., 2020), necroptosis (Yan et al., 2022), immunogenic cell death (Kroemer et al., 2022), entosis (Mlynarczuk-Bialy et al., 2020), methuosis (Maltese and Overmeyer, 2014), parthanatos (Zhou Y. et al., 2021), ferroptosis (Jiang X. et al., 2021), autosis (Nah et al., 2020a), alkaliptosis (Chen F. et al., 2023), oxeiptosis (Holze et al., 2018), cuproptosis (Xie J. et al., 2023), erebosis (Park et al., 2023), disulfidptosis (Liu X. et al., 2023) (Figure 1). RCD is initiated in mammalian cells upon irreparable disruption of intracellular or extracellular homeostasis, activating defined signaling cascades that execute programmed cellular elimination. Each RCD subtype operates through molecularly interconnected signaling networks with extensive crosstalk (Tong et al., 2022). These variants display a continuum of morphological phenotypes (ranging from necrotic to apoptotic) and immunomodulatory effects (spanning anti-inflammatory/tolerogenic to pro-inflammatory/immunogenic responses) (Peng et al., 2022). Distinct lethal subroutines within RCD pathways critically influence tumorigenesis and therapeutic outcomes. During early carcinogenesis, malignant cells frequently acquire chemoresistance via mutations disrupting core RCD machinery—a canonical cancer hallmark as per the oncogenic paradigm. Pharmacological modulation of RCD signaling, either singly or synergistically, may overcome therapeutic resistance in specific malignancies or combinatorial treatment regimens. Emerging evidence underscores the therapeutic potential of targeting RCD pathway crosstalk, offering novel strategies for precision oncology (Tong et al., 2022). This review traces the historical trajectory of RCD classification frameworks, interrogating subtype-specific morphological signatures and associated biochemical effectors. We consolidate cutting-edge discoveries in death-signaling network topology, with particular emphasis on pharmacologically actionable nodes and their clinical extrapolation. Through systematic integration of mechanistic paradigms with unmet clinical needs, this work establishes a blueprint for advancing therapeutic discovery through precision modulation of RCD pathway plasticity (Table 1).

**TABLE 1:** Characteristics of the major types of cell death.

Type of cell death	Triggers	Key molecules and phosphorylation	Molecular mechanisms	Morphological features	Cancer relevance
Apoptosis (Pascal et al., 1997; Thomas et al., 1995)	DNA damage, growth factor deprivation, death receptor activation (e.g., FasL/TRAIL)	Caspase-9 (Tyr153 phosphorylation, EGFR inhibition) BAD (Ser112/136 phosphorylation, AKT/PKA regulation) p53 (Ser15/20 phosphorylation, ATM/ATR activation)	death receptor/FasL → caspase-8 activation→ cytochrome C release from mitochondria → caspase-9/3activation → DNA breaks, nuclear fragmentation→ release of apoptotic vesicles	Cellular crumpling, chromatin condensation, nuclear fragmentation, apoptotic vesicle formation	Cancer inhibition: removal of abnormal cells Pro-cancer: apoptosis resistance leading to chemotherapy resistance (e.g., Bcl-2 overexpression)
Autophagic Cell death (Liu S. et al., 2023; Miller and Thorburn, 2021)	Nutritional deprivation, mTOR inhibition, ER stress	ULK1 (Ser317/777 phosphorylation-AMPK activation; Ser757 phosphorylation-mTOR inhibition) Beclin-1 (Ser93/96 phosphorylation, CDK1 inhibition of autophagy)	nutrient deficiency/mTOR inhibition → ULK1 complex activation→ autophagosome formation → lysosomal degradation →cytoplasmic vacuolization, no inflammation	Massive autophagic vesicle formation, cytoplasmic vacuolization	Dual action Early cancer suppression (removing damaged cells) Late-stage cancer promotion (maintaining tumor cell survival)
necroptosis (Liu et al., 2022a)	TNF-α, viral infections, caspase inhibition	RIPK1 (Ser166 autophosphorylated) RIPK3 (Thr231/Ser232 phosphorylated) MLKL (Thr357/Ser358 phosphorylated, RIPK3 activated)	TNF-α/Caspase inhibition → RIPK1-RIPK3 complex formation → MLKL phosphorylation and membrane perforation → cell swelling, membrane rupture → HMGB1/DAMPs release	Cell swelling, cell membrane rupture, organelle edema, content leakage	Pro-cancer: pro-inflammatory microenvironment supports metastasis Cancer suppression: activation of anti-tumor immunity (release of DAMPs)
pyroptosis (Vasudevan et al., 2023; He et al., 2015)	Pathogen infection, inflammatory vesicle activation (e.g., NLRP3)	NLRP3 (Ser198 phosphorylated, NEK7-regulated) ASC (Tyr144 phosphorylated, SYK-activated) Gasdermin D (cleavage-activated, non-phosphorylated*)	Pathogen/NLRP3 activation → Caspase-1 cleaves Gasdermin D →Cell membrane pore formation → IL-1β/IL-18 release, cell swelling	Cell membrane pore formation, cell swelling, nuclear consolidation, release of IL-1β/IL-18	Cancer inhibition: activation of anti-tumor immunity Pro-cancer: chronic inflammation promotes cancer (e.g., colon cancer)
ferroptosis (Tang et al., 2021)	Iron overload, GPX4 inhibition, lipid peroxidation	GPX4 (Tyr96 phosphorylation, SRC inhibitory activity) ACSL4 (Thr328 phosphorylation, MAPK-enhanced activity) Nrf2 (Ser40 phosphorylation, PKC-promoted entry)	GPX4 inhibition/iron overload →accumulation of lipid peroxidation → loss of mitochondrial cristae → loss of membrane integrity	Decreased mitochondrial cristae, lipid peroxidation accumulation, and membrane-free rupture	Cancer inhibition: killing drug-resistant tumor cells Pro-cancer: ferroptosis escape to promote metastasis (e.g., Hippo pathway activation)
Lysosomal cell death (Gómez-Sintes et al., 2016)	Lysosomal membrane permeabilizer (LLOMe), ROS	LAMP1 (ubiquitination-K63, lysosomal localization) Cathepsin B (self-shear activation) TFEB (phosphorylated-Ser142, inhibits lysosomal generation)	Lysosomal membrane permeabilization (LMP) → Cathepsin B release → Caspase activation/mitochondrial damage	Lysosomal rupture, cytoplasmic acidification	Oncogenic: induction of drug-resistant cell death Pro-cancer: TFEB hyperactivation promotes metastasis
Mitoptosis (Mijaljica et al., 2010; Lin et al., 2024)	Mitochondrial DNA damage, ROS overload	MTP18 (phosphorylated-Thr56, promotes mitochondrial division) DRP1 (ubiquitination-K48, proteasomal degradation)	MTP18 phosphorylation (Thr56) → DRP1 ubiquitination (K48 degradation) → mitochondrial division → mitochondrial autophagy	Fragmentation of the mitochondrial network and no membrane rupture	Dual action: removal of damaged mitochondria (cancer inhibition) → genomic destabilization (cancer promotion)
Paraptosis (Hanson et al., 2023)	IGF-1R activation, ER stress	ALG-2 (calcium binding, conformational change) HSP90 (acetylated-Lys294, inhibits client protein stabilization)	ALG-2/Ca^2+^ binding → MAPK/ERK activation → endoplasmic reticulum expansion → vacuolization death	Endoplasmic reticulum/mitochondria vacuolated, absence of apoptotic bodies	Pro-cancer: supporting tumor cell adaptation to stressful environments
NETosis (Vorobjeva and Chernyak, 2020; Zhu et al., 2022)	Pathogens, IL-8	PAD4 (citrullinated-histone H3 Arg8/17/26) NE (myeloperoxidase activation)	PAD4 activation → histone H3 citrullination (Arg8/17/26) → chromatin depolymerization → NE release → NETs formation	NETs formation, chromatin leakage	Pro-cancer: NETs promote the metastatic microenvironment
Immunogenic cell death (ICD) (Dai et al., 2023; Wang et al., 2024)	Anthracyclines, radiotherapy	CALR (exposed to cell surface, unmodified) HMGB1 (acetylated-Lys28/29, enhanced release)	ER stress → CALR surface exposure → HMGB1 acetylation (Lys28/29) release → DC activation → T cell response	Surface expression of CALR/HMGB1	Cancer inhibition: activation of anti-tumor immunity
Entosis (Durgan and Florey, 2018; Kim and Lee, 2014)	Cell extrusion, nest loss signaling	RhoA (phosphorylated-Ser188, inhibitory activity) E-cadherin (β-catenin binding, mediates cellular phagocytosis)	RhoA phosphorylation (Ser188, inhibition) → E-cadherin/β-catenin-mediated → nested cellular phagocytosis → lysosomal degradation	Nested cellular structures, involuntary phagocytosis	Dual Role: Clearing Tumor Cells vs Promoting Heterogeneity
Methuosis (Lin et al., 2020; Overmeyer et al., 2011)	Ras mutation, EGFR hyperactivation	RAC1 (ubiquitinated-K63, activates megaloblast drinking) Arf6 (phosphorylated-Tyr418, inhibits endosomal recycling)	Rac1 ubiquitination (K63) → macrophage activation → vesicle accumulation → mechanical rupture	Giant vesicle accumulation, nuclear extrusion	Cancer inhibition: targeting Ras mutant tumors
Parthanatos (Dorff et al., 2024)	DNA alkylating agent, PARP1 hyperactivation	PARP1 (PARylation-self-modification) AIF (cleavage release, unmodified)	PARP1 own PARylation → AIF cleavage release → nuclear DNA fragmentation	Nuclear consolidation, mitochondrial swelling	Promoting cancer: mechanisms of PARP inhibitor resistance
Autosis (Nah et al., 2020a)	Autophagic lysosomal overload, Na^+^/K^+^-ATPase inhibition	Na^+^/K^+^-ATPase (phosphorylated-Thr15, inhibits ion pumps) Rubicon (ubiquitination-K27, inhibits autophagosome maturation)	Rubicon ubiquitination (K27) → autophagosome maturation blockage → Na^+^/K^+^-ATPase phosphorylation (Thr15) → ion imbalance → membrane rupture	Autophagic vesicle fusion, nuclear membrane rupture	Controversial mechanism, possible cancer inhibition
Alkaliptosis (Song et al., 2018; Que et al., 2023)	High pH microenvironment	CA9 (deubiquitinated-K48, stabilizes carbonic anhydrase activity) SLC4A4 (phosphorylated-Ser982, inhibits HCO3- transport)	CA9 deubiquitination (K48) → H^+^ secretion blocked → intracellular alkalinization → membrane blistering	Swelling of cells, blistering of membranes	At the beginning of the study, potential cancer inhibitors
Oxeiptosis (Liu et al., 2021; Xuan et al., 2022)	High concentration of ROS	KEAP1 (ubiquitination-K48, degrades Nrf2) PGAM5 (dephosphorylated-Ser37, activates pro-death function)	KEAP1 ubiquitination (K48 degradation of Nrf2) → PGAM5 dephosphorylation (Ser37) → mitochondrial apoptosis	Nuclear consolidation, no inflammation	Cancer inhibition: removal of oxidatively damaged cells
Cuproptosis (Wang et al., 2023c; Kong and Sun, 2023)	Copper ion accumulation	FDX1 (copper binding induced conformational change) DLAT (lipoic acidation-dependent copper toxicity)	FDX1 copper binding → thioctylated protein aggregation (e.g., DLAT) → mitochondrial toxicity → ATP depletion	Mitochondrial protein aggregation, ATP depletion	Cancer inhibition: targeting tumors with abnormal copper metabolism
Erebosis (Ciesielski et al., 2022)	Continuous activation of the unfolded protein response	IRE1α (phosphorylated-Ser724, activates RNAase activity) XBP1 (spliced, unmodified)	IRE1α phosphorylation (Ser724) → XBP1 splicing → CHOP activation → pro-apoptotic gene expression	ER expansion, nuclear fragmentation	Unknown mechanism, possibly cancer-promoting
Disulfidptosis (Xiao et al., 2024; McDonnell et al., 1989)	Cystine starvation, SLC7A11 inhibition	SLC7A11 (ubiquitination-K63, lysosomal degradation) Nrf2 (acetylation-Lys599, enhanced transcriptional activity)	SLC7A11 ubiquitination (K63 degradation) → glutathione depletion → abnormal cross-linking of protein disulfide bonds → cell contraction	Abnormal cross-linking of disulfide bonds, cellular crumpling	Cancer inhibition: targeting antioxidant-deficient tumors

## 2 Programmed cell death

### 2.1 Apoptosis

**FIGURE 2:**
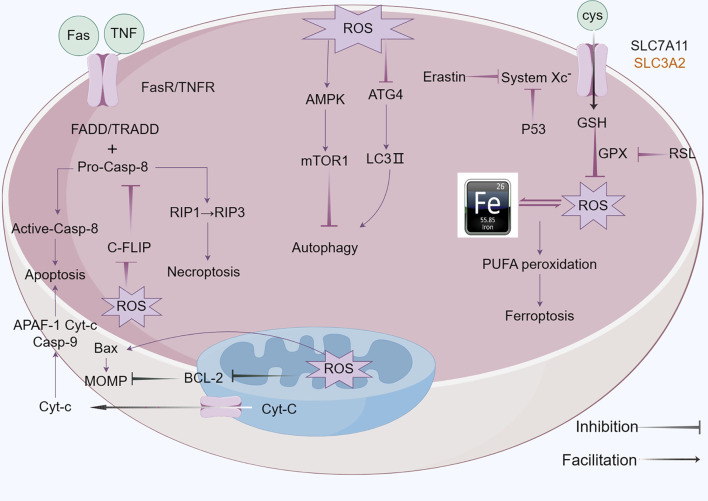
Mechanism of apoptosis,autophagy and ferroptosis: A There are two core apoptosis pathways, intrinsic and extrinsic. The extrinsic pathway is initiated by multiple death receptors, such as TNFR1, Fas, and DR4/5. The intrinsic pathway is mediated by Bcl-2 family proteins. Activation of either pathway ultimately triggers a cascade of caspases, thus inducing caspase-dependent nucleosome fragmentation leading to cell death. In addition, NF-κB, JAK-STAT3, and MAPKs signaling pathways play an essential role in regulating cell apoptosis; B Microtubule-associated protein one light chain 3 (LC3) undergoes lipidation (LC3-II) to promote autophagosome formation, a step controlled by autophagy-related gene 4 (Atg4). Inhibition of Atg4 stabilizes LC3-II, increasing autophagosome accumulation; C Ferroptosis is characterized by the depletion of intracellular glutathione and decreased activity of glutathione peroxidase 4 (GPX4), which leads to the accumulation of unmetabolized lipid peroxides and increased ROS production. Membrane damage is also a result of lipid peroxidation.

Apoptosis (Type I programmed cell death) constitutes a genetically encoded, actively regulated cell death mechanism governed by stringent molecular checkpoints (Peter et al., 1997). This process is principally executed through two canonical pathways: intrinsic (mitochondria-mediated) and extrinsic (death receptor-mediated) apoptosis, with emerging evidence for perforin/granzyme-mediated activation (Kashyap et al., 2021; Mustafa et al., 2024). The extrinsic pathway initiates upon tumor necrosis factor (TNF) superfamily receptor engagement (e.g., Fas/CD95), driving death-inducing signaling complex (DISC) assembly through sequential recruitment of death domain (DD)-containing adaptors (FADD/TRADD), death effector domain (DED) proteins, and procaspase-8 (Bodm et al., 2000; Schmitz et al., 2000). Caspase-8 undergoes autocatalytic activation within the DISC, initiating the executioner caspase cascade. This process is antagonized by cellular FLICE-inhibitory protein (c-FLIP) via competitive inhibition of caspase-8 recruitment and DISC stabilization (Pascal et al., 1997). Intrinsic apoptosis is activated by intracellular stressors (genotoxic damage, redox imbalance, metabolic crisis), culminating in mitochondrial outer membrane permeabilization (MOMP). This event is regulated by the Bcl-2 protein family hierarchy:Anti-apoptotic guardians (Bcl-2, Bcl-xL), Pro-apoptotic effectors (Bax, Bak) and BH3-only sensors (Bim, Bid, Puma) (Adams and Cory, 1998; Yang et al., 1997; Antonsson et al., 1997). In response to intracellular stress, the activation of pro-apoptotic BH3-only proteins counteracts the function of anti-apoptotic proteins, thereby enabling Bax and Bak to oligomerize and create pores in the mitochondrial membrane, facilitating the release of cytochrome c into the cytosol. The released cytochrome c interacts with apoptotic protease activating factor-1 (Apaf-1), facilitating apoptosome assembly and subsequent caspase-9 activation (Thomas et al., 1995) (Figure 2).

### 2.2 Autophagic cell death

Autophagic cell death (Type II programmed cell death) is driven by hyperactivation of autophagic machinery, characterized by cytoplasmic component sequestration into double-membrane autophagosomes for lysosomal degradation (Liu S. et al., 2023). While basal autophagy maintains cellular homeostasis via nutrient recycling and organelle quality control, pathological stressors (nutrient deprivation, oxidative stress, cytotoxic insults) can induce autophagic dysregulation culminating in cell death (Parzych and Klionsky, 2014). Three evolutionarily conserved autophagy subtypes are recognized: Macroautophagy: Non-selective engulfment of cytosolic cargo via autophagosome formation (Miller and Thorburn, 2021); Chaperone-mediated autophagy (CMA): Hsc70-dependent targeting of KFERQ motif-containing proteins to lysosomes through LAMP2A receptor recognition (Li et al., 2021); Microautophagy: Lysosomal membrane invagination-mediated direct engulfment of damaged organelles marked by DAMPs (Kim and Lee, 2014). In oncogenesis, ROS-mediated mechanisms critically regulate autophagic flux. ROS inactivates ATG4 cysteine protease activity to stabilize LC3-associated autophagosomes. Concurrently, oxidative stress activates the NRF2-p62/SQSTM1 axis and FOXO3 transcriptional network:NRF2 induces antioxidant genes and p62/SQSTM1 expression and FOXO3 upregulates LC3/BNIP3 to promote autophagosome-lysosome fusion. This coordinated response mitigates oxidative damage through enhanced cargo clearance. Autophagic initiation is governed by mTORC1-AMPK antagonism: AMPK phosphorylates and inhibits mTORC1 kinase activity, relieving its suppression on ULK1/ATG13 autophagy initiation complexes (Yamamoto et al., 2023) (Figure 2).

### 2.3 Autosis

**FIGURE 3:**
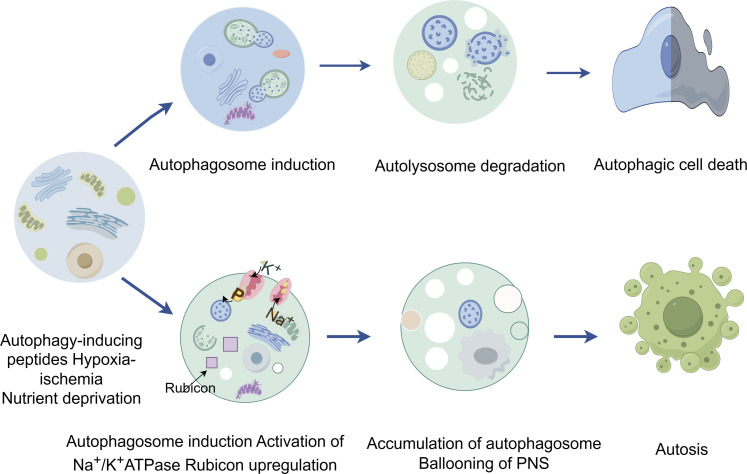
The figure highlights the contrast between autophagy and autosis, two processes involving autophagy. While autophagic cell death is a result of excessive autophagy, autosis is characterized by three distinct phases characterized by cells with unique morphological features and is triggered by various signals, such as Na^+^/K^+^-ATPase, Tat-Beclin 1, and hypoxia-ischemia.

Autosis, a unique autophagy-dependent cell death modality first identified in 2013 (Liu and Levine, 2015), is etymologically derived from the Greek *autos* (self) and *-osis* (pathological condition). Distinct from cytoprotective autophagy—a survival mechanism governed by mTORC1-AMPK-ULK1 signaling—autosis executes pathological cell death through Na^+^/K^+^-ATPase dysfunction (in Figure 3). Ultrastructural progression occurs in three phases: Initiation—proliferation of autophagosomes/autolysosomes, mitochondrial condensation, and dilated endoplasmic reticulum (ER) (Nah et al., 2020a; Jurgen and Ben, 2019). Intermediate—nuclear envelope separation with dense membrane domain formation; Terminal—perinuclear ballooning, mitochondrial swelling, and organelle disintegration. Mechanistically, Beclin 1-Na^+^/K^+^-ATPase interaction drives autophagic flux, with cardiac glycoside sensitivity serving as a diagnostic hallmark (Dep et al., 2024). Pathologically, autosis is inducible by Tat-Beclin1 *in vitro* and hypoxic-ischemic brain injury *in vivo*. Execution involves Rubicon-mediated blockade of autophagosome-lysosome fusion, causing ER/mitochondrial membrane depletion that precipitates organelle dysfunction—manifested by mitochondrial depolarization (ΔΨm loss) and ER structural collapse. Notably, Na^+^/K^+^-ATPase’s regulatory crosstalk with ATG proteins and ion homeostasis remains undefined, necessitating further exploration (Nah et al., 2020b; Tanaka et al., 2016) (Table 2).

**TABLE 2:** Core differences between autosis and autophagy.

Feature	Autophagy	Autosis
Functional Role	Cell survival mechanism (damage clearance, homeostasis maintenance)	Pathological demise (cell death due to autophagy hyperactivation)
Morphological Hallmarks	Autophagosome-lysosome fusion	Aberrant autophagosome accumulation, ER dilation, plasma membrane blebbing
Molecular Markers	LC3-II/p62 degradation	Downregulation of Na^+^/K^+^-ATPase α1 subunit (ATP1A1), elevated intracellular Na^+^ levels
Interventional Outcomes	Blocked by autophagy inhibitors (e.g., chloroquine)	Induced/alleviated by Na^+^/K^+^-ATPase inhibitors (e.g., digoxin)

### 2.4 Lysosomal cell death

**FIGURE 4:**
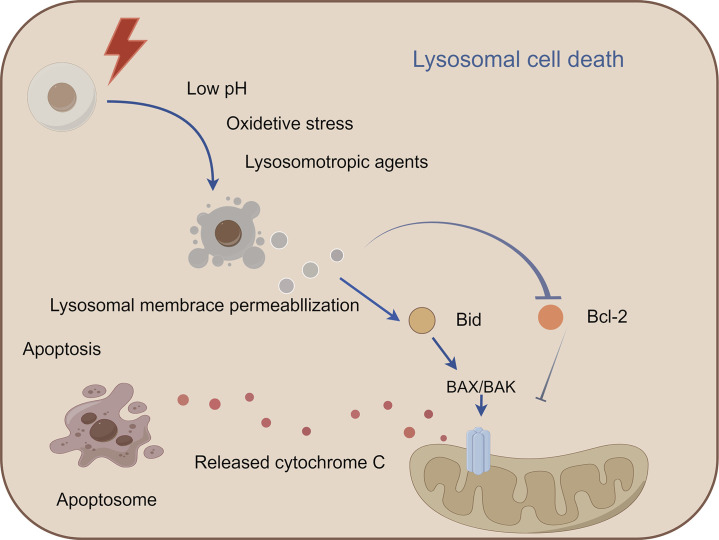
Mechanism of lysosomal cell death. This figure illustrates lysosomal cell death caused by lysosomal membrane permeabiliza-tion and the release of lysosomal enzymes into the cytoplasm, leading to the activation of apoptotic cell death pathways. Lysosomal cell death can be induced by stimuli, such as changes in lysosomal pH, oxidative stress, and lysosomotropic agents. The release of lysosomal proteases, such as cathepsins, activates the lysosomal apoptotic pathway by cleaving Bid and degrading antiapoptotic Bcl-2 homologs.

Lysosomal cell death (LCD), a regulated death modality initiated by lysosomal membrane permeabilization (LMP), is characterized by cytoplasmic release of cathepsins and activation of downstream death execution pathways (Yuan and Ofengeim, 2024). As central degradative compartments, lysosomes maintain cellular homeostasis through hydrolytic processing of intra- and extracellular substrates via pH-dependent enzymes (Gómez-Sintes et al., 2016). Pathological LMP triggers cathepsin-mediated cascades that converge on apoptosis (via Bid cleavage and Bcl-2 inactivation), necrosis, and ferroptosis (Aits and Jäättelä, 2013). Notably, cathepsin B/D activation establishes a proteolytic amplification loop, exacerbating mitochondrial outer membrane permeabilization and caspase activation (Aits and Jäättelä, 2013; Eriksson et al., 2020). Therapeutic exploitation of LCD demonstrates dual oncological relevance: (Galluzzi et al., 2018): Tumor-selective vulnerability: Cancer cells exhibit lysosomal hypertrophy and heightened LMP susceptibility due to metabolic reprogramming. (Grohmann et al., 2021). Resistance modulation: LCD bypasses classical apoptosis resistance mechanisms mediated by Bcl-2 overexpression or caspase mutations. Current LCD-targeting strategies (e.g., siramesine, lysosomotropic agents) face challenges in tumor specificity and systemic toxicity (Serrano-Puebla and Boya, 2018). Future directions should focus on: Developing organelle-specific delivery systems (e.g., pH-sensitive nanoparticles); Rational combination with immune checkpoint inhibitors or PARP antagonists, and Biomarker-driven patient stratification based on lysosomal gene expression signatures (Figure 4).

### 2.5 Mitoptosis

Mitoptosis (mitochondrial programmed cell death), first proposed by Skulachev in 1999 (Skulachev, 1999), s a ROS-driven cell death modality triggered by mitochondrial damage, with pathological implications spanning cancer, neurodegeneration, and metabolic diseases (Zong et al., 2016; Biswas et al., 2024). This process involves three sequential phases: (Galluzzi et al., 2018): mitochondrial network fragmentation and perinuclear aggregation, (Grohmann et al., 2021), membrane encapsulation forming mitoptotic bodies, and (Morana et al., 2022) extracellular extrusion via plasma membrane rupture (Mijaljica et al., 2010). Two mechanistically distinct subtypes exist: the inner membrane variant selectively degrades matrix/cristae while preserving outer membrane integrity, whereas the outer membrane variant induces cristae swelling followed by outer membrane rupture and cytoplasmic debris dispersa (Lin et al., 2024). Crucially, mitoptosis operates independently of autophagic machinery, contrasting sharply with mitophagy—a PINK1/Parkin-regulated process that eliminates damaged mitochondria through autophagosome-lysosome fusion (Wang S. et al., 2023). While both pathways maintain cellular homeostasis by clearing defective mitochondria, mitoptosis achieves this through self-encapsulation and extracellular expulsion, whereas mitophagy relies on lysosomal degradation. This dichotomy is further evidenced by their functional divergence: mitoptosis predominates in pathological clearance contexts, while mitophagy sustains basal mitochondrial quality control (Tinari et al., 2007).

### 2.6 Immunogenic cell death

**FIGURE 5:**
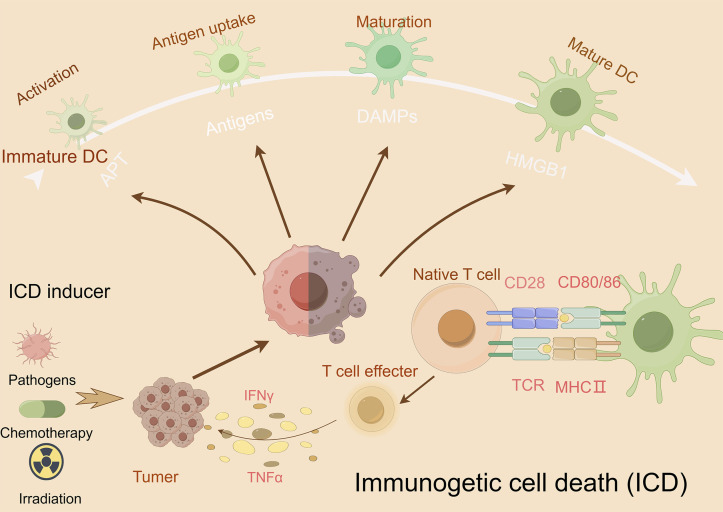
Mechanism underlying immunogenic cell death (ICD). This figure illustrates the mechanism of ICD and its potential as a cancer therapeutic strategy. During ICD, dying cells release damage-associated molecular patterns (DAMPs), such as ATP, high-mobility group box 1 (HMGB1), and heat shock proteins (HSPs), which activate dendritic cells (DCs) and other immune cells, promoting antigen presentation and immune activation. Effector T cells release interferon (IFN)-γ and TNFα, which activate other immune cells, such as natural killer cells and macrophages that detect and eliminate cancer cells.

Immunogenic cell death (ICD), first conceptualized by Kroemer and Zitvogel in 2005, represents a programmed cell death modality characterized by spatiotemporal release of damage-associated molecular patterns (DAMPs) (Casares et al., 2006). These endogenous danger signals—including surface-exposed calreticulin, secreted ATP, and released HMGB1—engage pattern recognition receptors (TLRs, NLRs) on antigen-presenting cells (APCs), triggering dual antitumor effects: direct neoplastic elimination and establishment of adaptive immune memory (Zhou et al., 2019) (Ahmed and Tait, 2020). Recent advances illuminate ICD’s translational potential through innovative therapeutic engineering: (Galluzzi et al., 2018): R848@M2pep-MPs AFP (Huazhong University): Macrophage-derived microparticles co-loaded with tumor antigens and TLR7/8 agonist R848 reprogram tumor-associated macrophages and expand stem-like CD8^+^ T cell clones, synergizing with anti-PD-1 therapy in hepatocellular carcinoma (Zhang et al., 2023; Bie et al., 2023). (Grohmann et al., 2021) cGAS-STING activation paradigm (Tsinghua-Harvard collaboration): Chemotherapy-induced tumor DNA release activates APC-intrinsic cGAS-STING signaling, establishing a molecular rationale for ICD-based combination strategies (Wang C. et al., 2023). These paradigm-shifting approaches demonstrate how precision-engineered ICD induction can overcome tumor immunosuppression, offering blueprint frameworks for next-generation immuno-oncology therapeutics (Figure 5).

### 2.7 Pyroptosis

**FIGURE 6:**
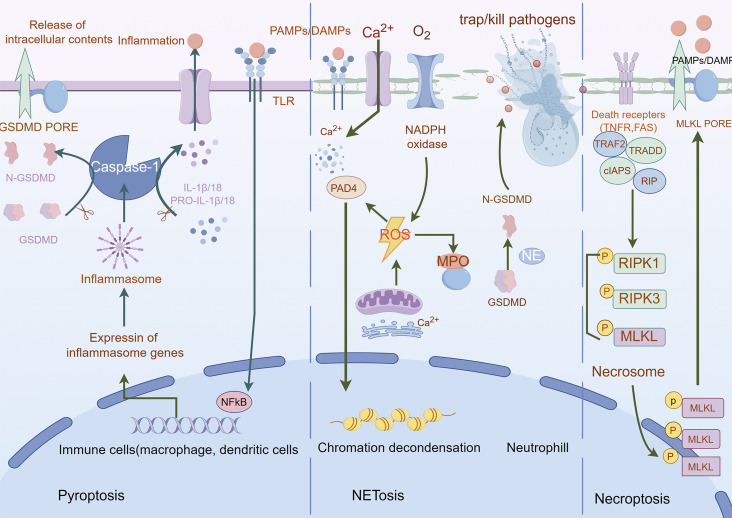
Mechanisms of pyroptosis, NETosis, and necroptosis. A Pyroptosis is characterized by cell swelling, plasma membrane rupture, and the release of proinflammatory cytokines, such as interleukin (IL)-1β and IL-18. Pyroptosis is triggered by the activation of inflammasomes, cytoplasmic complexes that sense danger signals, and initiate a caspase-1-dependent cascade that ultimately leads to cell death. B NETosis is a process in which neutrophils release DNA fibers coated with antimicrobial peptides to trap and kill pathogens. During NETosis, neutrophils undergo marked morphological changes, including chromatin decondensation, nuclear envelope rupture, and granule mixing, leading to the formation of neutrophil extracellular traps (NETs). The release of NETs is triggered by various stimuli, such as pathogens, cytokines, and immune complexes. C Necroptosis is mediated by death receptors. Upon activation of death receptors, such as TNFR1, receptor-interacting. Protein kinase 1 (RIPK1) binds to RIPK3 to form a necrosome. The necrosome complex promotes the oligomerization and phosphorylation of the mixed lineage kinase domain-like protein (MLKL). The oligomeric form of MLKL is translocated from the cytosol to the plasma membrane, leading to the formation of membrane pores and subsequent plasma membrane rupture. This results in the release of damage-associated molecular patterns (DAMPs), which trigger inflammation.

Pyroptosis—a lytic inflammatory cell death executed by gasdermin family protein-mediated nanoscale pore formation—releases pro-inflammatory cytokines (IL-1β/IL-18) to amplify immune responses, with its molecular basis established through the identification of GSDMD as the executioner via caspase-1-mediated proteolysis (Rao et al., 2022) (Vasudevan et al., 2023) (He et al., 2015). The gasdermin family (human: GSDMA-E, PJVK; murine: GSDMA-D, GSDME, PJVK) operates through autoinhibited structures requiring caspase/granzyme cleavage to liberate pore-forming N-terminal domains (Chen and Broz, 2024; Angosto-Bazarra et al., 2022), regulated by the Ragulator-Rag-mTORC1 axis for oligomerization and NINJ1 for membrane rupture, while ESCRT-III machinery counteracts progression via Ca^2+^-dependent repair (Broz et al., 2020) (Fang et al., 2020). In oncology, pyroptosis exhibits context-dependent duality: chronic activation (e.g., GSDME-driven HMGB1/ERK1/2 signaling in colitis-associated cancer) promotes tumorigenesis (Hu et al., 2024; Wei et al., 2022), whereas acute induction triggers immunogenic cell death through DAMPs and cytokines (IL-1β/IL-18) that enhance dendritic/NK cell activation and cytotoxic T cell priming, reshaping immunosuppressive microenvironments (Dai et al., 2023) (Wang et al., 2024) (Liu W. et al., 2023). Therapeutic exploitation focuses on spatiotemporal induction protocols, combinatorial regimens with immune checkpoint blockade, and biomarker-guided stratification to leverage pyroptosis’ immunomodulatory potential while mitigating pro-tumorigenic risks (Figure 6).

### 2.8 NETosis

NETosis, a neutrophil-specific lytic cell death modality first characterized by Brinkmann et al., in 2004 (Bont et al., 2019), mediates extracellular trap (NET) release—decondensed chromatin networks decorated with histones and antimicrobial proteins that entrap pathogens (Brinkmann et al., 2004). This process is initiated by NADPH oxidase-dependent ROS generation triggered by cytosolic Ca^2+^ elevation, progressing through three coordinated phases: (Galluzzi et al., 2018): nuclear envelope disintegration via neutrophil elastase (NE) and myeloperoxidase (MPO) released from azurophilic granules; (Grohmann et al., 2021); chromatin decondensation driven by peptidylarginine deaminase 4 (PAD4)-mediated histone citrullination (Thiam et al., 2020); and (Morana et al., 2022) GSDMD pore assembly activated by NE (non-canonical caspase-independent cleavage), enabling chromatin extrusion (Vorobjeva and Chernyak, 2020; Metzler et al., 2014; Ravindran et al., 2019). Organellar crosstalk underpins NETosis execution: endoplasmic reticulum (ER)-derived Ca^2+^ mobilizes NADPH oxidase complexes, while mitochondrial ROS amplifies oxidative signaling (Kambara et al., 2018; Gupta and Kaplan, 2016). In oncology, NETosis exhibits context-dependent duality—promoting metastasis through immunosuppressive NET deposition yet offering therapeutic vulnerabilities. PAD4 inhibition suppresses ovarian cancer peritoneal dissemination (Zhu et al., 2022). While DNase I-mediated NET degradation reverses chemoresistance in multiple myeloma (Chen XQ. et al., 2023). Current challenges include elucidating histone acetylation’s regulatory role and developing spatiotemporal modulation strategies to exploit NETosis’ antimicrobial benefits while curbing its pro-tumorigenic effects (Ravindran et al., 2019) (Figure 6).

### 2.9 Necroptosis

Necroptosis, a lytic programmed cell death modality distinct from apoptosis and necrosis, executes inflammatory demise through sequential activation of the RIPK1-RIPK3-MLKL signaling axis (Gao et al., 2022). Mechanistically, extracellular stimuli (FasR/TNFR1 engagement) or intracellular nucleic acid sensing (via TLR3/4 or ZBP1) trigger RHIM domain-mediated RIPK3 oligomerization, which phosphorylates MLKL to form membrane-disrupting necrosomes—pore complexes that induce plasma membrane permeabilization, cytoplasmic swelling, and organelle dysfunction (Kaczmarek et al., 2013) (Yuan et al., 2019; Samson et al., 2021; Seifert et al., 2016). This lytic process releases pro-inflammatory mediators (IL-1α, HMGB1) and potassium ions, amplifying immune activation through pattern recognition receptor signaling (Kaczmarek et al., 2013). In oncology, necroptosis exhibits context-dependent duality: while glucose deprivation induces tumor-suppressive necroptosis in breast cancer (Liu C. et al., 2022). RIPK3 overexpression drives cholangiocarcinoma/pancreatic carcinogenesis, and its downregulation correlates with poor acute myeloid leukemia outcomes, highlighting lineage-specific regulatory paradoxes (Baik et al., 2021). Pharmacological intervention via RIPK1 inhibitors (e.g., necrostatin-1) demonstrates therapeutic potential (Seifert et al., 2016; Koo et al., 2015; Seehawer et al., 2018), though challenges persist in balancing its tumoricidal effects against pro-metastatic risks. Future strategies require biomarker-guided spatiotemporal modulation to exploit necroptosis’ immunogenic properties while circumventing oncogenic adaptation mechanisms (Figure 6).

### 2.10 Cuprotosis

**FIGURE 7:**
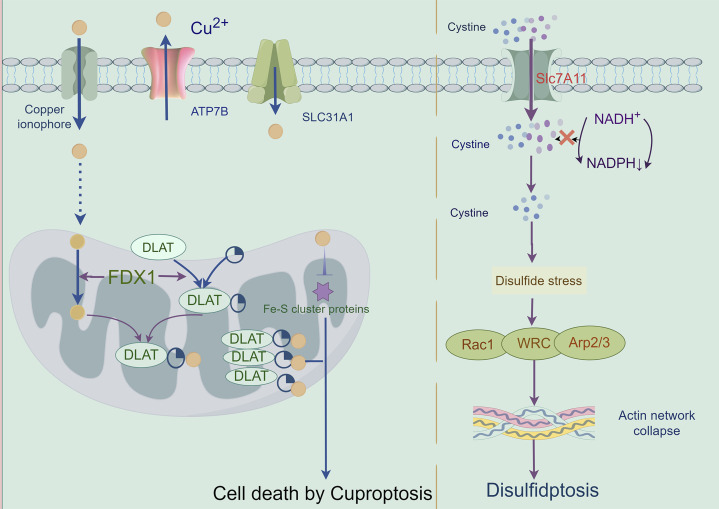
Mechanisms of cuproptosis and disulfidptosis: A cuproptosis is triggered by the accumulation of copper. It results in mitochondrial stress due to the aggregation of lipoylated mitochondrial enzymes and the loss of Fe–S cluster proteins, which can be mediated by ferredoxin 1 (FDX1). B When the NADPH supply is limited under glucose deprivation conditions, high cystine uptake by cells with high SLC7A11 expression results in intracellular NADPH depletion, the excessive accumulation of cystine and other disulfde molecules, and abnormal disulfde bond formation in actin cytoskeleton proteins, culminating in actin network collapse and disulfdptosis. Rac1-WRC-mediated branched actin polymerization and lamellipodia formation likely provide supporting conditions for disulfde bond formation in actin cytoskeleton proteins, thereby facilitating disulfdptosis.

Cuprotosis, a copper (Cu)-dependent regulated cell death pathway mechanistically distinct from apoptosis, necroptosis, pyroptosis, and ferroptosis, was formally characterized by Tsvetkov et al., in 2022 through functional interrogation of the copper ionophore elesclomol (Tsvetkov et al., 2022). This pathway exploits the elevated copper demand of malignant cells—a metabolic vulnerability linked to tumor progression and metastasis (Wang W. et al., 2023). Mechanistically, mitochondrial delivery of Cu^2+^ via ionophores (e.g., elesclomol) triggers FDX1-mediated reduction to Cu^+^, which induces proteotoxic stress through three convergent axes: (Galluzzi et al., 2018): lipoylated enzyme aggregation via direct Cu^+^ binding to TCA cycle components (e.g., DLAT); (Grohmann et al., 2021); Fe-S cluster destabilization in iron-sulfur proteins; and (Morana et al., 2022) redox imbalance from ROS overproduction. Crucially, cuprotosis resists inhibition by pan-caspase inhibitors, ferrostatin-1, necrostatin-1, or N-acetylcysteine, confirming its independence from canonical death pathways (Tsvetkov et al., 2022). Therapeutic potential was first demonstrated in multiple myeloma models, where elesclomol synergized with proteasome inhibitors by activating the mitochondrial Cu^2+^-ROS axis—revising prior attributions of cytotoxicity solely to lipid peroxidation (Kong and Sun, 2023). These findings position cuprotosis as a promising therapeutic paradigm for targeting copper-addicted malignancies (Figure 7).

### 2.11 Ferroptosis

Ferroptosis, an iron-dependent regulated cell death modality first characterized by Stockwell et al., in 2012 (Dixon et al., 2012), is defined by mitochondrial shrinkage, outer membrane rupture, and intact nuclei lacking chromatin condensation (Jiang X. et al., 2021). Mechanistically driven by iron-catalyzed lipid peroxidation, its execution involves two interdependent axes: (Galluzzi et al., 2018): glutathione (GSH) depletion through system Xc− (SLC3A2/SLC7A11) inhibition, disabling GPX4’s antioxidant function and (Grohmann et al., 2021) FSP1-CoQ10 axis suppression, compromising NAD(P)H-dependent lipid peroxidation defense (Tang et al., 2021; Li J. et al., 2020). Pharmacological induction is achieved via Class I (sorafenib/sulfasalazine) or Class II (RSL3) agents targeting these pathways (Mou et al., 2019). Membrane phospholipid peroxidation—primed by ACSL4-mediated PUFA esterification—is amplified through autophagy-dependent iron homeostasis regulation, notably NCOA4-mediated ferritinophagy (Li et al., 2022; Li et al., 2023; Zheng and Conrad, 2020). Therapeutically, ferroptosis exhibits selective lethality in malignancies with high PUFA membrane content (e.g., hepatocellular carcinoma), therapy-resistant phenotypes (e.g., clear cell RCC), and iron/redox metabolic dependencies. Current challenges center on targeting the FSP1-CoQ10 axis and optimizing spatiotemporal induction to circumvent adaptive resistance mechanisms, positioning ferroptosis as a promising paradigm for precision oncology (Bersuker et al., 2019; Chen et al., 2021; Park and Chung, 2019; Koppula et al., 2021) (Figure 2).

### 2.12 Paraptosis

**FIGURE 8:**
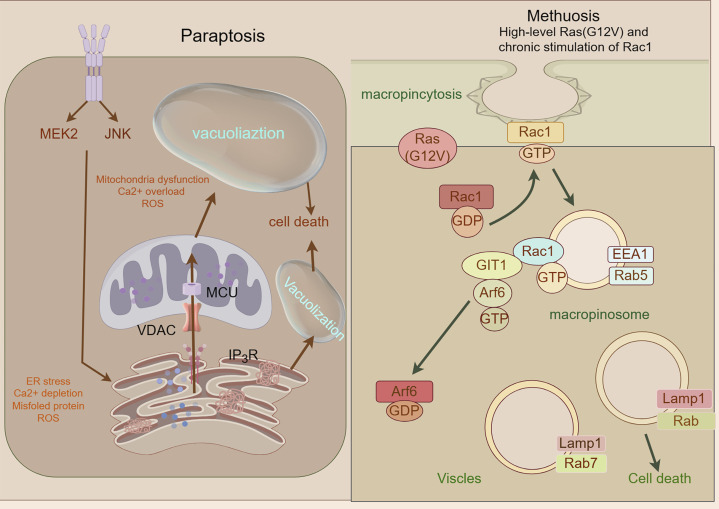
Mechanism underlying paraptosis and methuosis. A Paraptosis is character-ized by the development of large vacuoles in the endoplasmic reticulum (ER) and mitochondria, ultimately leading to the formation of large cytoplasmic vacuoles. Impaired proteostasis, altered ion homeostasis, and ER stress cause paraptosis, resulting in the discharge of Ca^2+^ from the ER and accumulation of Ca^2+^ in mitochondria. Paraptosis can be facilitated by the activation of mitogen-activated protein kinase (MAPK) signaling pathways via IGF-IR and inhibited by AIP-1/Alix; Molecular basis of methuosis. B Methuosis is initiated by prolonged high-level expression of RAS (G12V) and chronic activation of Rac1,which leads to enhanced macropinocytic activity. Moreover, this mechanism hampers macropinosome recycling by lowering the active Arf6 pool. Nascent macropinosomes, which are created from lamellipodial membrane projections, penetrate the cell and merge to form large fluid-filled vacuoles that, in contrast to typical macropinosomes, cannot be recycled. These vacuoles grow rapidly, resulting in a stable population with certain late endosomal features (Rab7 and LAMP1).

Paraptosis, a caspase-independent regulated cell death modality first identified in 2000 (Sperandio et al., 2000), is characterized by cytoplasmic vacuolization arising from endoplasmic reticulum (ER) and mitochondrial swelling (Chen et al., 2024). Its molecular framework involves MAPK/ERK/JNK pathway activation, ER stress-induced unfolded protein response (UPR) with BiP/CHOP regulation, and ROS-mediated HO-2/NPR/CO/BKCa channel cascades that drive calcium efflux, mitochondrial enlargement, and ionic dysregulation (Xu et al., 2024; Hanson et al., 2023; Shi et al., 2021). Key mediators including HMGB1, prohibitin, and Alix coordinate vacuole formation and execution. Therapeutically, paraptosis offers multimodal anticancer strategies: it overcomes chemoresistance through agents like curcumin (dual proteasome/mitochondrial Na^+^/Ca^2+^ exchange inhibition) (Lee et al., 2016; Yoon et al., 2014); synergizes with immunotherapy via PERK knockout-induced DAMPs release to enhance dendritic/T-cell activation (Nguyen et al., 2022); enables innovative drug design (e.g., Cu^2+^-responsive micelles generating proteasome inhibitors) (Mandula et al., 2022); enables innovative drug design (e.g., Cu^2+^-responsive micelles generating proteasome inhibitors) (Chen et al., 2002; Hoa et al., 2007) These approaches exploit paraptosis’ immunogenic features while bypassing apoptosis resistance, positioning it as a transformative paradigm for precision oncology (Figure 8).

### 2.13 Methuosis

Methuosis (from Greek *methuo*, “to drink to excess”), a non-canonical cell death modality characterized by cytoplasmic accumulation of macropinosome-derived vacuoles, was first described by Overmeyer et al., in 2008 (Overmeyer et al., 2008). Mechanistically driven by synergistic activation of RasG12V and Rac1 GTPase signaling (Bhanot et al., 2010), this process involves pathological hyperactivation of macropinocytosis—a bulk endocytic pathway for extracellular solute uptake—coupled with impaired Arf6-dependent vesicle recycling (Lin et al., 2020). The resulting giant vacuoles coalesce, displacing organelles and ultimately causing plasma membrane rupture through volumetric stress (Overmeyer et al., 2011). While methuosis exhibits pan-cancer relevance in preclinical models, its molecular circuitry (e.g., vacuole-lysis triggers, stress-sensor involvement) remains incompletely mapped. Systematic characterization of its signaling architecture and therapeutic vulnerabilities is imperative for clinical translation (Figure 8).

### 2.14 Entosis

**FIGURE 9:**
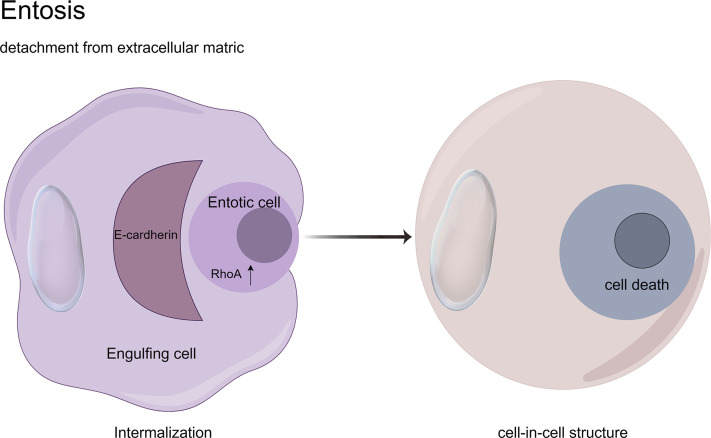
Cell-in-cell structures: a hallmark of entosis. Entosis is a biological process characterized by the internalization of one living cell into the cytoplasm of another. It is caused by adherent cell matrix separation, which results in the establishment of E-cadherin-mediated cell connections (shown in red) between the engulfing cell and the entotic cell. RhoA activity within the entotic cell causes actomyosin buildup at the cell cortex, resulting in the creation of cell-in-cell structures that mimic an active invasion-like process. Most internalized cells die as a result of entotic cell death, which is followed by lysosome fusion or apoptosis, especially when macroautophagy has been inhibited. However, certain entotic cells may divide within their hosts or even escape death.

Entosis, a non-apoptotic cell death mechanism characterized by cell-in-cell (CIC) engulfment, was first described by Overholtzer in 2007 (Overholtzer et al., 2007). This process initiates upon extracellular matrix detachment, where RhoA-ROCK1/2 signaling drives actomyosin contractility to mechanically invade neighboring cells. Mechanistically, E-cadherin/β-catenin complexes stabilize adhesion junctions while DIAPH1-mediated actin polymerization and Aurora kinase-regulated microtubule dynamics orchestrate membrane budding (Durgan and Florey, 2018). The internalized cell undergoes autophagy-dependent degradation via LC3-associated phagocytosis (LAP) within entotic vacuoles, distinct from classical apoptosis (Krishna and Overholtzer, 2016). Paradoxically, entosed cells may exhibit non-lytic egress or intracellular replication, suggesting context-dependent survival plasticity (Kim et al., 2024). Crucially, MRTF/SRF transcriptional reprogramming and ezrin-mediated membrane remodeling coordinate this cannibalistic process, positioning entosis as a unique interplay of mechanical forces and molecular signaling in tumor microenvironments (Gaptulbarova et al., 2024; Liu D. et al., 2020; Didan et al., 2024) (Figure 9).

### 2.15 Parthanatos

**FIGURE 10:**
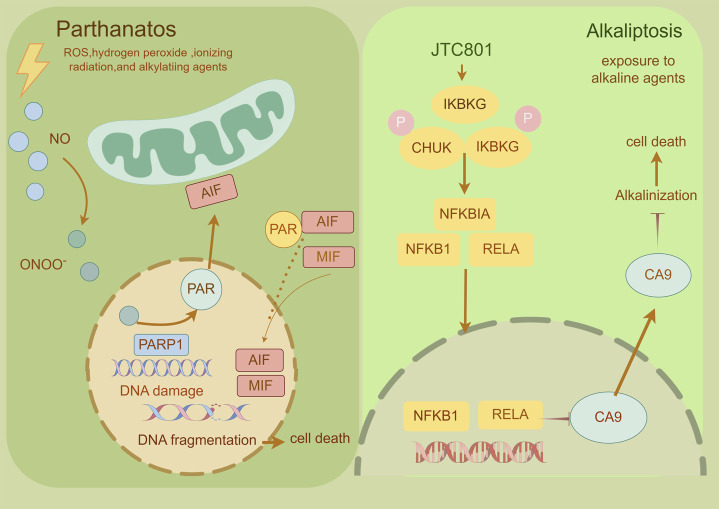
Mechanism underlying parthanatos and alkaliptosis. A This diagram depicts the molecular processes underlying parthanatos. ROS, ischemia, alkylating chemicals, and radiation activate PARP-1 by activating NOS, resulting in the creation of excess NO and subsequent synthesis of peroxynitrite (ONOO−). Peroxynitrite activates PARP-1, resulting in the formation of copious amounts of PAR polymer in the nucleus. Certain poly (ADP)-ribosylated carrier proteins escape from the nucleus, prompting the outer mitochondrial membrane to release apoptosis-inducing factor (AIF). AIF then enters the cytoplasm and attaches to macrophage migration inhibitory factor (MIF). AIF and MIF enter the nucleus and cause widespread DNA degradation, ultimately resulting in cell death. B This figure illustrates the activation mechanism of alkaliptosis, which is characterized by intracellular alkalinization and subsequent cell death. JTC801 activates the IKK protein complex, which includes CHUK (IKKα), IKBKB (IKKβ), and IKBKG (IKKγ). Then, the IKK protein complex phosphorylates and degrades NFKBIA (IκBα), leading to the nuclear translocation of NFKB1 (p50) or RELA (p65), which regulate gene expression. Furthermore, NF-κB negatively regulates the expression of CA9, a member of the carbonic anhydrase family, to inhibit alkaliptosis.

Parthanatos, a caspase-independent regulated necrosis pathway driven by PARP-1 hyperactivation, is defined by three hallmarks: (Galluzzi et al., 2018): cytotoxic poly (ADP-ribose) (PAR) polymer accumulation, (Grohmann et al., 2021), mitochondrial bioenergetic collapse enabling apoptosis-inducing factor (AIF) nuclear translocation, and (Morana et al., 2022) chromatin fragmentation without canonical apoptotic/necrotic features (Fatokun et al., 2014; Liu L. et al., 2022; Yijie et al., 2019; Zheng et al., 2022). Distinct from apoptosis (lacking DNA laddering), necrosis (no organellar edema), or necroptosis (RIPK1-independent), this pathway executes cell death through PAR-mediated metabolic catastrophe and AIF-driven 15–50 kb DNA cleavage. Therapeutically, PARP-1 inhibitors (Olaparib, Rucaparib) have achieved clinical success in BRCA-mutant breast/ovarian cancers, while combinatorial regimens (e.g., Olaparib + carboplatin + pembrolizumab) demonstrate enhanced progression-free survival (Matulonis et al., 2016; Mirza et al., 2016; Swisher et al., 2017; Litton et al., 2018; Pujade-Lau et al., 2017). Emerging strategies targeting PARG (BZL101), MIF-CD74 (ISO-1), and AIF nuclear shuttling are expanding therapeutic reach, with ongoing trials (NCT04821622) evaluating Talazoparib in metastatic prostate cancer (Feng et al., 2012; Dorff et al., 2024). By exploiting PAR-mediated DNA damage amplification and bypassing apoptosis resistance mechanisms, parthanatos modulation represents a paradigm-shifting approach for solid tumor therapy (Figure 10).

### 2.16 Alkaliptosis

Alkaliptosis, a pH-dependent regulated cell death pathway activated under alkaline conditions (pH > 8.0), was first conceptualized by Tang et al., in 2018 as a therapeutic strategy against apoptosis-resistant malignancies (Song et al., 2018). Mechanistically, NF-κB pathway activation induces transcriptional repression of carbonic anhydrase 9 (CA9) through p65/RelA nuclear translocation and H3K4me3-mediated chromatin remodeling, while ACSS2-mediated acetyl-CoA production sustains NF-κB activation via histone acetylation (Tang et al., 2019; Liu J. et al., 2020; Que et al., 2023). This dual regulatory loop establishes a self-reinforcing alkaline tumor microenvironment by elevating cytosolic pH, particularly in pancreatic ductal adenocarcinoma (PDAC) models. Therapeutically, alkaliptosis exploits cancer cells’ pH dyshomeostasis—a hallmark of metabolic reprogramming linked to proliferation and metastasis. Pharmacological modulation of this pathway (e.g., NF-κB/ACSS2 inhibition) demonstrates potential to circumvent chemoresistance, though challenges remain in delineating its molecular circuitry (e.g., pH-sensing mechanisms, CA9-independent variants) and optimizing pH-modulating agents for clinical translation (Liu J. et al., 2020; Bulusu et al., 2017) (Figure 10).

### 2.17 Oxeioptosis

**FIGURE 11:**
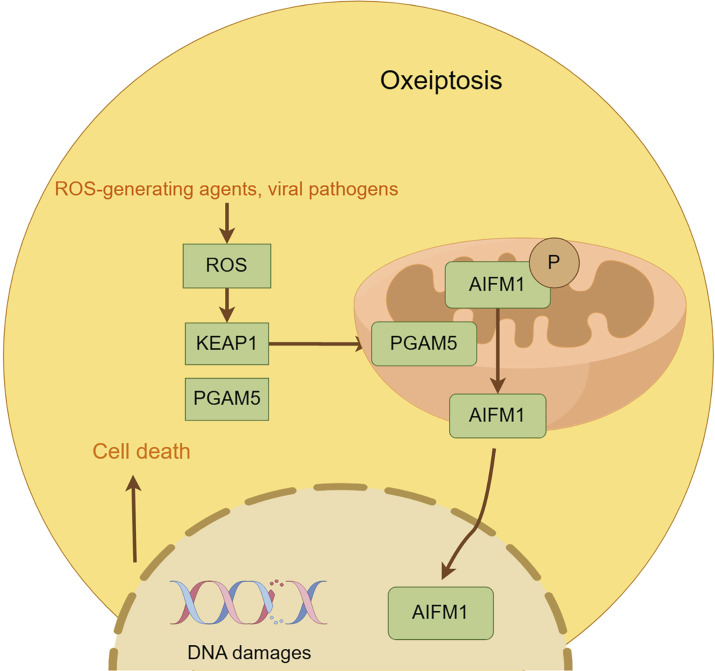
Mechanism underlying oxeiptosis. This figure illustrates the key features of oxeiptosis. Oxeiptosis is activated in response to oxidative stress induced by ROS or ROS-generating agents, such as viral pathogens. The KEAP1/PGAM5/AIFM1 signaling pathway plays a central role in oxeiptosis, in which AIFM1 is dephosphorylated under oxidative stress conditions via the regulatory action of PGAM5. Dephosphorylated AIFM1 is translocated from mitochon-dria to the nucleus, leading to.

Oxioptosis, a caspase-independent, ROS-driven cell death modality first identified by Holze et al., in 2018, is orchestrated by the KEAP1-PGAM5-AIFM1 axis under oxidative stress (e.g., H_2_O_2_, ozone). Mechanistically, KEAP1 senses ROS to release Nrf2 for nuclear antioxidant gene activation while simultaneously triggering PGAM5 mitochondrial translocation, which dephosphorylates AIFM1 at Ser116, inducing mitochondrial permeabilization without nuclear translocation (Holze et al., 2018; Liu et al., 2021; Lin et al., 2023). This pathway exhibits context-dependent duality: pro-death in colorectal cancer via Auriculasin-enhanced KEAP1/AIFM1 signaling *versus* pro-survival upon KEAP1/AIFM1 inhibition (Wang et al., 2021; Chang et al., 2023). Therapeutic exploitation is challenged by endogenous antioxidants (e.g., GPX), while its pathological role extends to melanocyte loss in vitiligo through AIFM1 dephosphorylation (Rosini and Pollegioni, 2022). These findings position oxioptosis as a redox-sensitive death switch with dual therapeutic implications—leveraging its cytotoxicity against resistant malignancies while mitigating oxidative tissue damage (Xuan et al., 2022) (Figure 11).

### 2.18 Erebosis

In 2022, Yoo’s research team discovered a unique cell death pathway, designated Erebosis, in adult *Drosophila* intestinal epithelial cells. Erebosis is defined by the gradual degradation of the cytoskeleton, cell adhesion molecules, organelles, and DNA, culminating in cell death. This phenomenon is localized to the R4 region of the intestine, where cell turnover is most pronounced, and is prevalent in intestinal stem cells and younger epithelial cells. Ance proteins accumulate in cells undergoing Erebosis, although their precise function remains to be elucidated. Erebosis diverges from classical cell death pathways (apoptosis/necroptosis/autophagy) through its unique morphological and molecular signatures: intact plasma membranes without lytic rupture, lack of chromatin condensation or apoptotic body formation, and a distinct proteolytic profile bypassing caspase/calpain-mediated cleavage cascades. Cells undergoing Erebosis are ultimately detected by TUNEL staining. The researchers propose that Erebosis may serve as a unique cell death mechanism that facilitates the continuous renewal of intestinal tissues while maintaining tissue integrity and avoiding immune responses (Ciesielski et al., 2022).

### 2.19 PANoptosis

PANoptosis, a tri-modal cell death program integrating pyroptosis, apoptosis, and necroptosis, was first conceptualized by Kanneganti et al., in 2019 based on inflammasome studies during influenza infection (Shi et al., 2023). This process is driven by PANoptosome assembly—a supramolecular complex comprising ZBP1 (vRNP sensor), RIPK1/RIPK3 (necroptosis executors), NLRP3/ASC/caspase-1 (pyroptosis mediators), and caspase-8 (apoptosis initiator)—which synergistically coordinates membrane permeabilization and cytokine storm generation (Gao et al., 2024). Mechanistically, ZBP1 activates PANoptosis through dual roles: structural recognition of viral ribonucleoproteins and scaffolding of caspase-6-mediated PANoptosome oligomerization (Zheng and Kanneganti, 2020). Pathogenically, *Yersinia* YopJ effector disrupts TAK1-mediated inhibitory phosphorylation of RIPK1/RIPK3, enabling TLR/death receptor-driven PANoptosome assembly (Malireddi et al., 2023). Execution involves caspase-1/8-mediated cleavage of GSDMD (pyroptotic pore formation) and RIPK3 (necroptosis propagation), amplifying immunogenic cell death (Wang L. et al., 2023; Samir et al., 2020). While implicated in COVID-19 pathophysiology, the existence of dedicated phagocytic complexes in PANoptosis remains unconfirmed, necessitating further mechanistic validation to resolve this critical knowledge gap.

### 2.20 Disulfidptosis

Disulfidptosis, a novel disulfide stress-driven cell death modality identified by Liu et al., in 2023 (Gu et al., 2024), is characterized by pathological actin cytoskeletal collapse via SLC7A11-mediated cystine overload under glucose deprivation. Mechanistically, SLC7A11-overexpressing malignancies accumulate intracellular cystine, generating excessive disulfide bonds that crosslink actin filaments through Rac-WAVE regulatory complex (WRC)-dependent lamellipodial remodeling (Li T. et al., 2024). This process destabilizes membrane-cytoskeleton interactions, culminating in cell death. Disulfidptosis induction extends beyond glucose limitation to include H2O2 exposure and TXNRD2 inhibition, though its onset is attenuated in SLC7A11-low cells, suggesting crosstalk with apoptotic/necroptotic pathways (McDonnell et al., 1989). Therapeutically, disulfidptosis exploits metabolic vulnerabilities in SLC7A11-high tumors: while SLC7A11 confers resistance to ferroptosis/apoptosis, its overexpression creates glucose dependency, rendering cells susceptible to GLUT inhibitors—as demonstrated in KEAP1-mutant lung cancers where SLC7A11 upregulation drives disulfide accumulation under glucose starvation (Xiao et al., 2024). Current challenges include the absence of specific biomarkers and targeted inhibitors, necessitating preclinical validation of disulfidptosis-inducing agents to advance precision oncology strategies for SLC7A11-driven malignancies (Liu X. et al., 2023) (Figure 7) (Table 3).

**TABLE 3:** *In vivo* experiments in the mode of cell death.

Compounds	Subjects (cells/animals)	Concentration	Research mechanisms	Main mechanisms	Tumor type	References
rTLM-PEG + PTX	Balb/c nude mice (A549/T drug-resistant NSCLC model)	rTLM-PEG: 2 mg/kg PTX liposomes: 2 mg/kg	Evaluated the inhibitory effect of rTLM-PEG combined with PTX liposomes on drug-resistant tumors; monitored tumor growth, body weight changes, and apoptosis markers (cleaved caspase 3, TUNEL staining)	rTLM-PEG is activated by MMP-2 to release CPP-TCS, inhibits PTX-induced caspase 9 phosphorylation, activates caspase 3-dependent apoptosis, and enhances PTX efficacy	Drug-resistant non-small cell lung cancer (A549/T cells)	Chen et al. (2017a)
BTSA1	NOD-SCID IL2Rγ null (NSG) mice + THP-1 cells	10 mg/kg (IP every 48 h)	Evaluated BTSA1’s effect on survival in AML xenografts; analyzed leukemia infiltration and apoptosis markers (e.g., caspase-3 cleavage, TUNEL staining)	Direct BAX activation, inducing AML apoptosis and suppressing leukemia growth	Acute Myeloid Leukemia (AML)	Reyna et al. (2017)
γ-PGA/PEI/pTRAIL NPs	10 µg pTRAIL/mice (peritumoral injection every 2 days)	10 µg pTRAIL/mice (injected peritumorally every 2 days)	To evaluate the inhibitory effect of γ-PGA/PEI/pTRAIL nanoparticles on cervical cancer tumor growth; to monitor changes in body weight, tumor volume, and to conduct histopathological analysis	γ-PGA enhances tumor cell uptake through GGT-mediated endocytosis, inhibits TRAIL-induced apoptosis, and significantly suppresses tumor growth	Cervical cancer (HeLa cells)	Tan et al. (2017)
AMG 176 + Venetoclax	MOLM-13 xenografted mice (AML model)	AMG 176: 30 mg/kg (q48h, oral) Venetoclax: 50 mg/kg (daily, oral)	Dual inhibition of MCL1 and BCL-2 Synergistic induction of apoptosis	The combined use of AMG 176 and Venetoclax significantly inhibits tumor growth, achieving complete suppression of tumor burden	Acute Myeloid Leukemia (AML)	Caenepeel et al. (2018)
Venetoclax、GDC-0980、Taselisib	NOD/SCID-γ mice (inoculated with MV4-11 AML cells)	Venetoclax: 80 mg/kg GDC-0980: 5→10 mg/kg Taselisib: 1.5–2.5 mg/kg	Combined inhibition of BCL-2 and PI3K/mTOR pathways, evaluating AML growth suppression and survival extension	BAX-dependent mitochondrial apoptosis; PI3K/mTOR inhibition leading to MCL-1 downregulation	Acute Myeloid Leukemia (AML)	Rahmani et al. (2018)
Dihydroartemisinin (DAT)	6-8-week-old female athymic nude mice (Foxn1^nu^ Foxn1^+^) inoculated with GPX4-inducible knockout H292 lung cancer cells (3 × 10^6^ cells/mouse)	DAT: 5 mg/kg (intraperitoneal injection) Liproxstatin-1: 10 mg/kg (intraperitoneal injection)	1. DAT induces lysosomal degradation of ferritin, increasing intracellular free iron levels 2. DAT disrupts the IRP-IRE signaling pathway, enhancing sensitivity to ferroptosis 3. DAT synergizes with GPX4 knockout to overcome intrinsic resistance to ferroptosis in tumor cells	1. Iron homeostasis regulation: DAT increases intracellular free iron, promoting lipid peroxidation 2. IRP-IRE signaling pathway: DAT binds to free iron, disrupting iron homeostasis feedback regulation 3. GPX4 inhibition synergy: DAT enhances GPX4 knockout-induced ferroptosis	Lung cancer (H292 cells)	Che et al. (2020)
DHA (Dihydroartemisinin)	PANC1, SW1990 cells	20–45 μM (PANC1), 40–120 μM (SW 1990)	Inhibition of cell proliferation, induction of DNA damage, disruption of mitochondrial homeostasis, induction of ferroptosis	Induces ferroptosis by modulating iron metabolism, enhances cisplatin cytotoxicity	Pancreatic Ductal Adenocarcinoma (PDAC)	Du et al. (2021)
Ce6 (Chlorin e6)	B16F10 melanoma cells and Panc02 pancreatic cancer cells	2.5 mg/kg	Inhibits tumor growth through photodynamic therapy (PDT), enhances immune response, and suppresses PD-1/PD-L1 immune checkpoint	Enhances CD8^+^ T cell activity and induces immunogenic cell death by inhibiting PD-1/PD-L1 interaction	Melanoma and Pancreatic Cancer	Gurung et al. (2023)
GSDMD Agonist (C1/C2) + anti-PD-1	C57BL/6 mice (MC38 colon cancer model)	C1: 10 mg/kg + anti-PD-1 (10 mg/kg) C2: 10 mg/kg + anti-PD-1 (10 mg/kg)	1. Combination therapy enhances antitumor effects 2. Enhances CD8^+^ T cell infiltration 3. Inhibits tumor growth and recurrence	1. GSDMD agonist synergizes with anti-PD-1 to activate immune response 2. Enhances T cell-mediated tumor killing	Colon cancer	Fontana et al. (2024)
HNE (4-hydroxynonenal)	C57BL/6 mice (acute lung injury model)	6 µM	Inhibits NLRP3 inflammasome activation, reduces inflammatory response and pyroptosis	Directly binds to NLRP3, inhibits its interaction with NEK7, reduces IL-1β release and pyroptosis	Acute Lung Injury	Hsu et al. (2022)
PPCNPs-Ce6/FA	Nude mice (MCF-7/ADR tumor model)	20 µM (equivalent Ce6 concentration)	Promotes cellular uptake of photosensitizers through nanocarrier delivery and folate targeting, selectively accumulates in lysosomes, triggers ROS generation with 660 nm laser, reduces P-gp expression, and enhances the chemotherapeutic efficacy of DOX	Promotes cellular uptake of photosensitizers through nanocarrier delivery and folate targeting, selectively accumulates in lysosomes, triggers ROS generation with 660 nm laser, reduces P-gp expression, and enhances the chemotherapeutic efficacy of DOX	Drug-resistant breast cancer	Li et al. (2016)
ALA-PDT	SKH-1 mice (SCC tumor model)	0.5 mM ALA, 0.5 J/cm^2^	Induces the expression of DAMPs (CRT, HSP70, HMGB1) through ALA-PDT, promotes phenotypic and functional maturation of dendritic cells (DCs), and enhances anti-tumor immune responses	ALA-PDT induces the expression of DAMPs, promotes DC maturation and anti-tumor immune responses, and enhances the immunogenicity of tumor cells	Cutaneous Squamous Cell Carcinoma (SCC)	Wang et al. (2015)

## 3 The role of regulated cell death (RCD) in cancer treatment

### 3.1 Induction of cell death as a standard practice in chemotherapy and radiotherapy for cancer treatment

Most cancer treatment methods discovered through empirical research, including alkylating agents, antimetabolites, topoisomerase inhibitors, and anti-microtubule compounds, primarily function through DNA synthesis inhibition, DNA damage induction, or replication interference (DeVita and Chu, 2008). Prolonged exposure of cancer cells to these substances at adequate concentrations can prevent them from replicating DNA and dividing, even if they are not completely eradicated. Unfortunately, these therapeutic agents also demonstrate significant cytotoxicity toward normal proliferating cells, particularly hematopoietic and gastrointestinal progenitor populations. Consequently, conventional chemotherapy and radiotherapy often feature a narrow therapeutic index and are limited by severe side effects. For numerous malignancies, current therapeutic doses remain inadequate for complete tumor eradication and patient cure. Extensive research over the past 30 years has revealed that sublethal doses of chemotherapy and radiotherapy can trigger tumor cell death through indirect mechanisms. Interestingly, a subset of cancer cells undergo apoptosis in response to drug-induced cellular stress rather than direct cytotoxicity (Vaux and Häcker, 1995; Xu et al., 2005; Kültz, 2005). Apoptotic commitment is regulated by a dynamic balance: Pro-apoptotic drivers include transcriptional activation (e.g., p53-mediated BIM induction) or post-transcriptional stabilization (e.g., miRNA-dependent PUMA regulation) of BH3-only proteins. Survival suppressors such as BCL-2/Bcl-xL sequester BH3 activators (BID, BAD), blocking mitochondrial outer membrane permeabilization (MOMP) and conferring therapy resistance (Herr and Debatin, 2001; Tsujimoto, 1989; McDonnell et al., 1989; Strasser et al., 1991; Men et al., 2021). The concurrent deletion of BAX and BAK (Lindsten et al., 2000), or the absence of BH3-only proteins (especially PUMA, BIM, or NOXA), can confer resistance to a variety of anticancer drugs in both malignant and non-transformed cells, including traditional chemotherapeutics (e.g., glucocorticoids, cyclophosphamide, and paclitaxel) and molecularly targeted agents (such as imatinib, a BCR-ABL inhibitor for chronic myeloid leukemia [CML] treatment) (Bouillet et al., 1999; Villunger et al., 2003; Kuroda et al., 2006; Jeffers et al., 2003). Numerous malignant cells exhibit genetic alterations, particularly p53 mutations, that compromise BH3-only protein expression, essential for drug-induced apoptotic signaling (Roos and Kaina, 2006; Vo and Letai, 2010). The on-target toxicity of conventional chemotherapeutics stems from their inability to discriminate between transformed and normal proliferating cells. Mitochondrial-mediated apoptotic pathways are activated in both malignant clones and sensitive normal compartments (e.g., intestinal crypt stem cells, granulocyte-macrophage progenitors), driving dose-limiting gastrointestinal and myelosuppressive toxicities (Hu et al., 2019). This phenomenon partially accounts for chemotherapy- and radiotherapy-induced toxicity in normal tissues (e.g., gastrointestinal mucosa and hematopoietic system). These treatments primarily target proliferating cells, regardless of their malignant status. Their efficacy in bone marrow stems from the presence of quiescent stem cells that resist treatment and subsequently proliferate to promote tissue regeneration. Developing strategies to selectively protect normal tissues from treatment-induced apoptosis would represent a major therapeutic breakthrough, potentially enabling more aggressive treatment protocols and improved remission rates.

### 3.2 Death receptor-induced apoptosis as a promising anticancer modality

Genetic studies indicate that apoptosis triggered by TNFR family death receptors is pivotal in conferring resistance to chemotherapeutic agents (Bukowski et al., 2020). These receptors initiate programmed cell death in cancer cells via the extrinsic apoptosis pathway, which serves to suppress tumor growth. Nonetheless, this process may inadvertently harm healthy cells, thereby constraining its clinical utility (Sartore-Bianchi et al., 2016). Despite this, death receptor therapy remains a promising avenue in the fight against cancer. For instance, recombinant human TRAIL protein has demonstrated cytotoxicity against a diverse array of cancer cell types, including melanoma, breast, and colorectal cancers, in both laboratory and *in vivo* settings (Fox and MacFarlane, 2016). However, the therapeutic potential of TRAIL protein is curtailed by its brief half-life in the body. To circumvent this limitation, researchers have advanced TRAIL gene therapy, which involves delivering the TRAIL gene directly to cancer cells to prolong its therapeutic impact. Furthermore, innovative death receptor agonists, such as the antibiotic Monensin, have been identified to potentiate TRAIL-mediated apoptosis, offering fresh strategies for overcoming cancer cell resistance (Chen et al., 2017a; Tan et al., 2017; Chen et al., 2017a). To enhance the therapeutic efficacy and safety profile of death receptor therapy, future research should focus on integrating death receptor treatments with other therapeutic modalities. For example, the concurrent administration of death receptor agonists and BH3 mimetic drugs (such as BCL-xL inhibitors) could synergistically activate both the extrinsic and intrinsic apoptosis pathways, thereby potentiating the killing of cancer cells. Moreover, the development of targeted drug delivery systems is imperative to ensure that therapeutic agents selectively target cancer cells, thereby minimizing off-target effects on healthy tissue (Tan et al., 2017).

In conclusion, death receptor therapy stands as a pioneering approach in the field of cancer treatment, brimming with untapped potential. By delving deeper into its mechanisms and investigating its synergistic potential with other treatments, we aspire to forge ahead with the development of more potent and safe cancer treatment strategies, offering a beacon of hope to cancer patients worldwide.

### 3.3 Targeting BCL-2 family proteins: innovative strategies for directly activating BAX/BAK in cancer treatment

Apoptosis, the primary mechanism of cell death in cancer cells, is meticulously regulated by BCL-2 family proteins (Hanahan and Weinberg, 2011; Singh et al., 2019). In recent years, these proteins, especially BCL-2 itself, have risen to prominence as pivotal targets for cancer therapy. The direct activation of BAX and BAK to elicit apoptosis in cancer cells is emerging as a highly promising therapeutic approach (Singh et al., 2019). For instance, BTSA1, a known activator of BAX, has showcased promising antitumor activity in acute myeloid leukemia (AML) cells and is currently under investigation for its potential synergistic effects when combined with other drugs, aiming to enhance its efficacy and combat resistance (Reyna et al., 2017). Moreover, the scientific community is actively exploring novel direct activators of BAX, such as S55746, and evaluating their antitumor potential across various cancer types. These innovative compounds hold the promise of circumventing the inhibitory effects of anti-apoptotic proteins, thereby facilitating a more potent induction of apoptosis in cancer cell (Casara et al., 2018). Furthermore, the synergistic combination of BCL-2 inhibitors with MCL-1 inhibitors, exemplified by AMG 176 and TP-1165, has demonstrated the potential to significantly impair the survival of cancer cells and overcome resistance to BCL-2 inhibitors (Caenepeel et al., 2018). Similarly, the conjunction of BCL-2 inhibitors with PI3K inhibitors, such as idelalisib and copanlisib, has shown the ability to inhibit the growth and survival of cancer cells, addressing the issue of resistance to BCL-2 inhibitors (Roberts et al., 2016; Rahmani et al., 2018). These avant-garde strategies have the capacity to circumvent the inhibitory effects of anti-apoptotic proteins, leading to a more effective induction of apoptosis in cancer cells. The continuous exploration of new pharmacological agents and combination treatment regimens is paving the way for the development of more efficacious and safer cancer treatment modalities, offering a beacon of hope to cancer patients worldwide.

### 3.4 The functional significance of alternative programmed cell death modalities in oncogenesis and therapeutic interventions

Programmed cell death, a genetically regulated active cellular termination process, plays an essential role in the development and maintenance of homeostasis in multicellular organisms and can be classified into apoptotic and non-apoptotic modalities. The induction of apoptotic pathways represents a fundamental therapeutic strategy in oncology, given the critical role of apoptotic regulation in both tumorigenesis and tumor suppression (Tan et al., 2009). The dysregulated overexpression of pro-survival BCL-2 in malignancies subverts mitochondrial apoptosis to drive oncogenic progression, whereas restoration of pro-apoptotic BCL-2 family effectors (BAX/BAK) reinstates mitochondrial outer membrane permeabilization (MOMP) to execute tumor-suppressive clearance (Kerr et al., 1972). Advances in multi-omics technologies have systematically decoded the molecular architecture of non-apoptotic cell death over the past 3 decades, identifying five major paradigms: (Galluzzi et al., 2018): ferroptosis, an iron-catalyzed lipid peroxidation cascade initiated by GPX4 suppression; (Grohmann et al., 2021); necroptosis, a kinase-driven necrotic pathway mediated by RIPK3-dependent MLKL oligomerization and plasma membrane rupture; (Morana et al., 2022); pyroptosis, an inflammasome-activated lytic process executed by caspase-cleaved gasdermin family proteins forming cytotoxic pores; (Krysko et al., 2012); paraptosis/lysosome-dependent death, marked by ER/mitochondrial vacuolization or lysosomal cathepsin leakage; and (Troitskaya et al., 2022) autophagic cell death, resulting from ATG5/7-mediated autophagosome overaccumulation and self-digestive organelle degradation. These discoveries have revealed unique signaling cascades and molecular mechanisms distinct from classical apoptosis, offering promising therapeutic targets and innovative strategies for cancer therapy (Strasser et al., 2011).

Ferroptosis therapy, as an emerging cancer treatment strategy, has demonstrated tremendous potential, particularly in overcoming the limitations of traditional chemotherapeutic drugs, showcasing its unique advantages (Dixon et al., 2012). For instance, RSL3, a well-characterized ferroptosis inducer, has demonstrated promising therapeutic efficacy in clinical trials for hepatocellular carcinoma (HCC). Experimental studies have revealed that RSL3 effectively induces hepatocellular carcinoma cell death and suppresses tumor progression, thereby offering a novel therapeutic approach for HCC management (Sui et al., 2018). Additionally, arabinoside, as a natural compound, has also been proven to induce ferroptosis in hepatocellular carcinoma cells and enhance the efficacy of chemotherapeutic drugs (Che et al., 2020). These findings demonstrate the extensive therapeutic potential of ferroptosis in oncology. Notably, dimethyl sulfoxide (DMSO) and oxaliplatin have been shown to trigger ferroptotic cell death in colorectal and lung cancer cells, effectively eliminating malignant cells and potentiating the effects of conventional chemotherapeutic agents (Du et al., 2021; Sato et al., 2018). Similarly, Tegafur and Cisplatin have demonstrated the capacity to initiate ferroptosis in pulmonary carcinoma cells, effectively eliminating malignant cells and potentiating the therapeutic effects of conventional chemotherapeutic regimens (Zhou S. et al., 2021). Additionally, sulfasalazine and olaparib have been found to induce ferroptosis in head and neck cancer cells, effectively killing head and neck cancer cells and inhibiting tumor growth (Yoshikawa et al., 2013). Pramipexole and azacitidine have similarly demonstrated ferroptosis-inducing capabilities in pancreatic carcinoma cells, effectively eradicating malignant cells and augmenting the efficacy of standard chemotherapeutic protocols. The therapeutic potential of ferroptosis extends across diverse malignancies, necessitating future investigations into targeted delivery systems for ferroptosis inducers, synergistic treatment modalities, and dosage optimization to enhance therapeutic outcomes while minimizing adverse effects, thereby facilitating the clinical translation of ferroptosis-based therapies. Additionally, the combination of ferroptosis with other treatment modalities has also shown great potential. For instance, the combination of ferroptosis with photothermal therapy can significantly enhance the therapeutic effect. For example, Fe_3_O_4_ nanoparticles can generate a photothermal effect under light irradiation, promoting tumor cell membrane permeability, enhancing the penetration and action of ferroptosis inducers, and thus effectively killing tumor cells (Li Y. et al., 2024). Photodynamic therapy (PDT) generates ROS that can interact with the lipid peroxidation process in the ferroptosis pathway, further promoting lipid peroxidation and enhancing the effect of ferroptosis (Kojima et al., 2024). For example, Ce6, as a photosensitizer, generates ROS under light irradiation, which acts synergistically with ferroptosis inducers to effectively kill tumor cells (Li H. et al., 2024). Immunomodulatory Effects: Ferroptosis exhibits immunostimulatory properties by activating innate and adaptive immune responses. Specifically, RSL3-mediated ferroptosis triggers the release of DAMPs, subsequently activating T lymphocytes and dendritic cells, thereby enhancing anti-tumor immunity. This immunogenic cell death mechanism demonstrates synergistic effects with immune checkpoint blockade, potentially improving therapeutic outcomes in cancer immunotherapy (Hao et al., 2024). These combination treatment strategies are expected to further enhance the efficacy of ferroptosis therapy, bringing new hope to cancer treatment.

As a RIPK1/RIPK3/MLKL axis-driven programmed necrosis, necroptosis is gaining traction in cancer therapeutics through its bifunctional mechanisms: (Galluzzi et al., 2018): direct tumoricidal effects via plasma membrane permeabilization, and (Grohmann et al., 2021) indirect immune activation through HMGB1/ATP release that enhances dendritic cell cross-presentation and CD8^+^ T cell infiltration (Rucker et al., 2024). For instance, key inhibitors targeting the RIPK1/RIPK3 signaling pathway, such as necrostatin-1 and GSK-3, have exhibited potent necroptosis-inducing capabilities in malignant cells (Zhang et al., 2024). Moreover, the synergy of necroptosis with photodynamic therapy (PDT) leverages reactive oxygen species generated by PDT to further exacerbate cell membrane damage, thereby amplifying the necroptotic effect (Gurung et al., 2023). A case in point is Ce6, a photosensitizer that under light irradiation generates ROS, acting in concert with necroptosis inducers to potently eliminate tumor cells (Chen et al., 2019). Additionally, necroptosis can also stimulate immune activation and enhance anti-tumor immunity. For instance, necroptosis inducer RSL3-mediated ferroptosis triggers DAMP release, subsequently activating T lymphocytes and dendritic cells, thereby potentiating anti-tumor immunity, and synergize with immune checkpoint inhibitors to improve treatment efficacy (Rucker et al., 2024). However, necroptosis therapy also faces challenges, such as cytotoxicity and tumor heterogeneity. Some necroptosis inducers may have a certain level of toxicity to normal cells, requiring further optimization of drug design and dosage to reduce toxicity (Wang et al., 2015). Furthermore, necroptosis can activate the immune system, bolstering anti-tumor immune responses. For example, the necroptosis inducer RSL3 can release DAMPs, thereby activating T cells and dendritic cells, potentiating anti-tumor immunity, and demonstrating synergistic effects with immune checkpoint blockade, can significantly improve treatment efficacy. Despite these advancements, necroptosis therapy confronts challenges such as cytotoxicity and tumor heterogeneity. Certain necroptosis inducers may exhibit toxicity toward normal cells, necessitating further refinement in drug design and dosage to mitigate this concern. Additionally, the heterogeneity of tumor cells in their sensitivity to necroptosis demands further investigation into strategies to augment the targeting and selectivity of necroptosis therapy. Nonetheless, as an innovative cancer treatment strategy, necroptosis therapy harbors significant potential. Future investigations should focus on developing targeted delivery systems for necroptosis inducers, establishing synergistic therapeutic regimens, and optimizing dosage parameters to maximize therapeutic efficacy while minimizing adverse effects, thereby propelling the clinical application of necroptosis therapy (Rucker et al., 2024).

Pyroptosis, a novel non-apoptotic cell death pathway, has shown tremendous promise in cancer treatment, with several clinical applications and recent breakthroughs. For example, doxorubicin induces pyroptosis in pulmonary carcinoma cells through caspase-1-mediated GSDMD proteolytic cleavage., enhancing the efficacy against drug-resistant tumors (Fontana et al., 2024). Furthermore, IFN-g secreted by CD8^+^ T cells can inhibit the Xc^−^ system, promote lipid peroxidation, and sensitize tumor cells to pyroptosis, thereby enhancing immunotherapy’s effectiveness (Hsu et al., 2022). Recent advancements include the development of new pyroptosis inducers, such as berberine and artemisinin derivatives, which induce pyroptosis through diverse signaling pathways and mechanisms, offering higher selectivity and lower toxicity (Wang et al., 2020; Jiang et al., 2021b). Nanodrug delivery systems can precisely target pyroptosis inducers to tumor sites, minimizing damage to normal cells. For example, targeted nanoparticles can deliver pyroptosis inducers directly to tumor cells, improving treatment efficacy while reducing side effects (Li et al., 2016). Additionally, the integration of pyroptosis induction with conventional therapies, including chemotherapy, photodynamic therapy, and immunotherapy, demonstrates synergistic therapeutic effects and improved clinical outcomes. Photodynamic therapy, for instance, can generate reactive oxygen species, promoting pyroptosis and thereby boosting anti-tumor effects (Li W. et al., 2020). Future efforts should focus on developing personalized pyroptosis induction strategies, delving deeper into the molecular mechanisms and signaling pathways of pyroptosis, and assessing the safety of pyroptosis inducers to advance their clinical application in cancer treatment.

### 3.5 Immunotherapy

Immunotherapy represents a revolutionary therapeutic strategy that harnesses the host’s immune system to recognize and eradicate malignant cells, revolutionizing the cancer therapy landscape (Xie N. et al., 2023) (Table 4). Immuno-oncology advances are propelled by three paradigm-shifting modalities—immune checkpoint disruption (anti-PD-1/CTLA-4) to reverse T-cell exhaustion, genetically engineered CAR-T cells targeting CD19/BCMA for tumor-specific cytotoxicity, and dendritic cell vaccines loaded with neoantigens to prime antigen-presenting cells—which collectively overcome tumor immune evasion mechanisms, achieving durable remission rates exceeding 50% in refractory lymphomas (NCT02348216). As a monoclonal antibody targeting the PD-1/PD-L1 axis, nivolumab (Opdivo) reinvigorates exhausted T cells, demonstrating durable clinical responses across multiple tumor types—melanoma (objective response rate [ORR] 40%–45%), NSCLC (ORR 20%–30%), and RCC (ORR 25%–35%)—with manageable immune-related adverse events (irAEs) (Socinski et al., 2018). Tisagenlecleucel (Kymriah), a CAR-T therapy, has demonstrated significant clinical effectiveness in specific subtypes of ALL (Davila et al., 2014). The integration of immunotherapy with cytotoxic modalities (chemotherapy/radiotherapy) creates synergistic antitumor effects through two mechanisms: 1) chemotherapeutic agents trigger immunogenic cell death (ICD) via autophagy/pyroptosis/ferroptosis pathways, and 2) released DAMPs (HMGB1/ATP) and cytokines (IL-1β/IFN-γ) activate dendritic cells and enhance CD8^+^ T-cell infiltration, overcoming tumor-mediated immunosuppression. This dual-action strategy enhances treatment depth by simultaneously eradicating tumor cells and establishing long-term immune surveillance (Galon and Bruni, 2019; Zhang et al., 2021). Radiotherapy can activate DNA sensing pathways, like STING and AIM2, triggering an inflammatory response and immunogenic cell death (ICD) (McLaughlin et al., 2020). These treatments can work synergistically to more effectively eradicate cancer cells and enhance immunotherapy’s efficacy. As research progresses, immunotherapy will become more effective, safer, and more economical, offering renewed hope to cancer patients.

**TABLE 4:** Comparative analysis of cancer therapeutic modalities.

Therapeutic modality	Advantages	Limitations	Applicable scenarios	Synergistic strategies	References
Chemotherapy/Radiotherapy	Broad efficacy (multiple tumor types) Established protocols Strong synergy potential (combination therapy)	Nonspecific toxicity (myelosuppression, gastrointestinal damage) Drug resistance mechanisms (ABC transporters/PARP upregulation) Genomic instability risks (secondary mutations)	Rapid control of advanced cancers (leukemia, lymphoma) Neoadjuvant/adjuvant therapy (tumor reduction or residual clearance)	Chemotherapy + immunotherapy (antigen release) Radiotherapy + targeted therapy (local control)	Gottesman et al. (2002), Weiss et al. (2016)
Death Receptor-Induced Apoptosis	Targeted activation (DR4/DR5 selectivity) Immune synergy (DAMPs release)	Receptor heterogeneity (epigenetic silencing/mutations) Poor pharmacokinetics (short TRAIL half-life) Off-target toxicity (hepatic injury)	DR4/DR5-high solid tumors (breast cancer, NSCLC) Combination therapy (BH3 mimetics)	TRAIL + BCL-2 inhibitors (navitoclax) Death receptor agonists + immune checkpoint inhibitors	Kang et al. (2019), Huang et al. (2016)
BCL-2 Family Targeting	Precision intervention (mitochondrial apoptosis regulation) Clinically validated (>80% response in CLL)	Lineage dependency (low solid tumor response) Compensatory resistance (MCL-1/BCL-xL upregulation) - Dose-limiting thrombocytopenia (BCL-xL inhibitors)	BCL-2-dependent hematologic malignancies (CLL, AML) Combination with MCL-1/PI3K inhibitors	Venetoclax + MCL-1 inhibitors (S63845) - BCL-2 inhibitors + chemotherapy (mitochondrial stress)	Roberts et al. (2016)
Non-Apoptotic PCD Pathways	Bypasses apoptosis resistance (effective in p53-mutant tumors) Immune activation (DAMPs release via pyroptosis/necroptosis)	Mechanistic complexity (ferroptosis relies on lipid peroxidation) Inflammatory toxicity (cytokine storm) Off-target effects (non-specific inducers)	Apoptosis-resistant solid tumors (TNBC, pancreatic cancer) - Conversion of “cold” to “hot” tumors	Ferroptosis inducers + photothermal therapy (Fe3O4 nanoparticles) Pyroptosis inducers + PD-1 inhibitors	Stockwell et al. (2017), Shi et al. (2015)
Immunotherapy	Durable responses (immune memory) High specificity (CAR-T antigen targeting) Broad applicability (solid/hematologic tumors)	Immunosuppressive microenvironment (Tregs/MDSCs) Toxicity (irAEs/CRS) High cost (CAR-T: >$500,000 per dose)	High tumor mutational burden (melanoma, MSI-H CRC) Hematologic malignancies (B-ALL, myeloma)	PD-1 inhibitors + chemotherapy (antigen release) CAR-T + epigenetic modulators (enhanced persistence)	Sharma et al. (2021), June et al. (2018)

## 4 Conclusion and future perspectives

**FIGURE 12:**
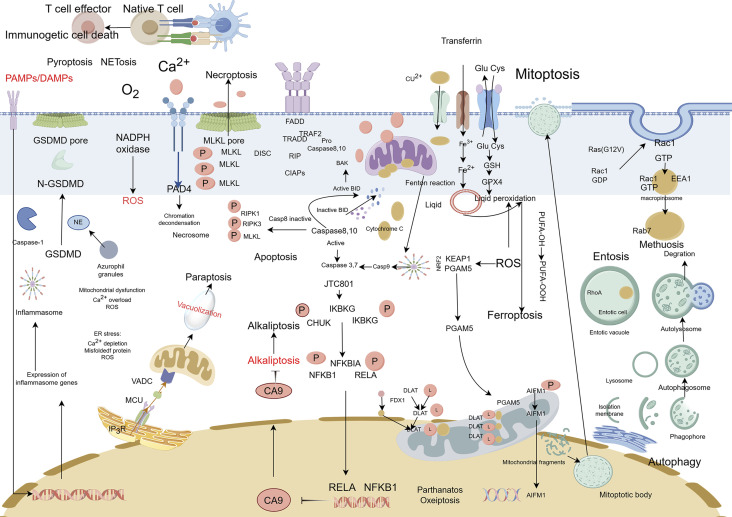
Complexity of cell death. This figure illustrates the complex and interconnected nature of cell death pathways. The figure shows the mechanisms by which different types of cell death pathways interact and influence each other and the ways in which they can be regulated by various signaling pathways and environmental factors.

The mechanisms of cell death, encompassing over 20 distinct forms including apoptosis, autophagic cell death, lysosomal-dependent death, paraptosis, pyroptosis, NETosis, necroptosis, and immunogenic cell death, have unveiled revolutionary avenues for cancer therapeutics (Figure 12). These pathways exhibit diverse therapeutic potential through unique molecular drivers—such as lipid peroxidation in ferroptosis and disulfide stress in disulfidptosis—and their synergistic integration with conventional therapies (chemotherapy/radiotherapy) and emerging immunotherapies is reshaping oncological treatment paradigms. However, clinical translation faces three pivotal challenges: First, off-target toxicity, exemplified by hepatotoxicity from the ferroptosis inducer RSL3 (targeting ubiquitously expressed GPX4) and compromised tissue repair via necroptosis inhibitors (e.g., Necrostatin-1); Second, tumor heterogeneity, manifested as intertumoral variability in death pathway activation (e.g., SLC7A11 expression gradients influencing disulfidptosis susceptibility between primary and metastatic lesions); Third, biomarker deficiencies, where current indicators (e.g., malondialdehyde for lipid peroxidation) lack spatiotemporal resolution, necessitating advanced dynamic imaging probes (e.g., FRET-based disulfide bond sensors).

To address these barriers, dual strategies are prioritized: Therapeutically, precision combination regimens are being engineered, such as ferroptosis inducers (e.g., Erastin) combined with anti-PD-1 immunotherapy to activate STING-dependent CD8^+^ T-cell recruitment (validated in the NCT04379855 trial for non-small cell lung cancer); Scientifically, multidimensional classification frameworks are evolving to integrate NCCD genetic criteria, metabolic signatures (e.g., intracellular GSH/GSSG ratios), and microenvironmental features defined by spatial transcriptomics, enabling prediction of dominant death pathways. Through technological innovations (CRISPR screening for resistance targets, real-time death process monitoring via nanoprobes) and adaptive clinical trial designs (basket trials stratified by ACSL4 activity), the vision of “on-demand death induction” tailored to individual molecular profiles is advancing, heralding a new era of precision oncology with transformative potential for global cancer care (Table 5).

**TABLE 5:** Chemical compounds and their structural information.

Compound name	Structure	Mechanism of action	Associated cell death type/Therapeutic modality	References
Elesclomol	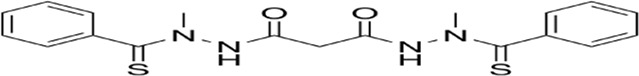	Copper ionophore; induces mitochondrial Cu^2+^ accumulation	Cuprotosis	Wang et al. (2023c)
RSL3	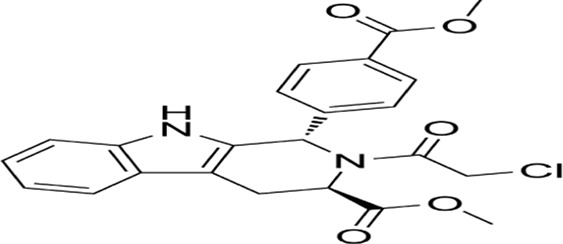	GPX4 inhibitor; induces lipid peroxidation	Ferroptosis	Li et al. (2022)
Olaparib	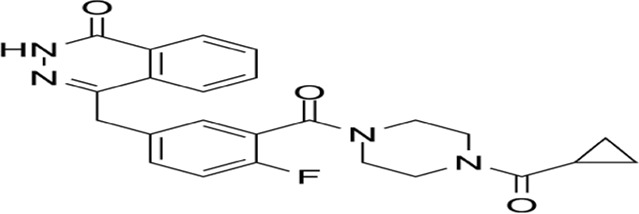	PARP1 inhibitor; synthetic lethality in BRCA-mutant cancers	Parthanatos	Matulonis et al. (2016)
Curcumin	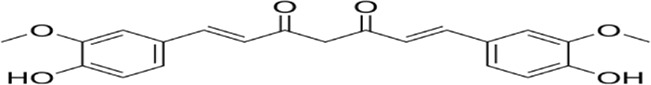	Dual inhibition of proteasome and mitochondrial Na^+^/Ca^2+^ exchange	Paraptosis	Nguyen et al. (2022)
Bortezomib	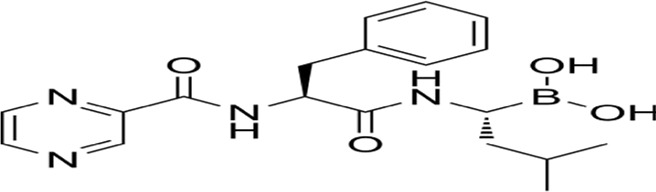	Proteasome inhibitor; synergizes with paraptosis inducers	Paraptosis	Mandula et al. (2022)
Necrostatin-1	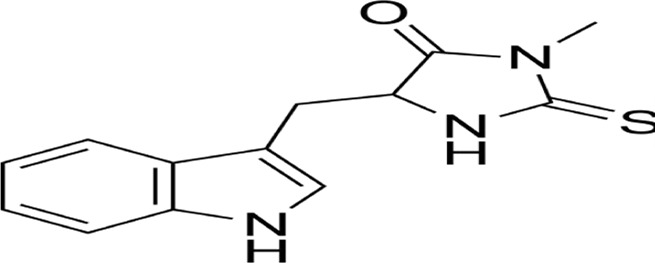	RIPK1 inhibitor; blocks necroptosis execution	Necroptosis	Baik et al. (2021), Zhang et al. (2024)
Ferrostatin-1	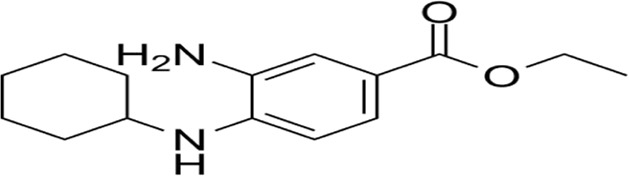	Lipid peroxidation inhibitor; suppresses ferroptosis	Ferroptosis	Li et al. (2022)
Monensin	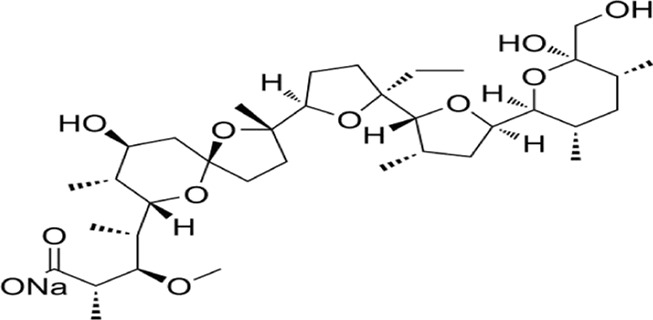	Antibiotic; enhances TRAIL-mediated apoptosis	Extrinsic Apoptosis	Chen et al. (2017a)
BTSA1	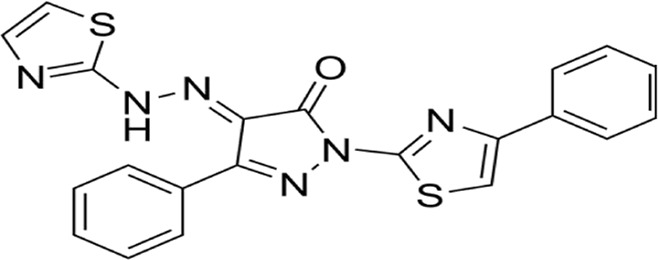	BAX activator; directly triggers mitochondrial apoptosis	Mitochondrial Apoptosis	Reyna et al. (2017)
S55746	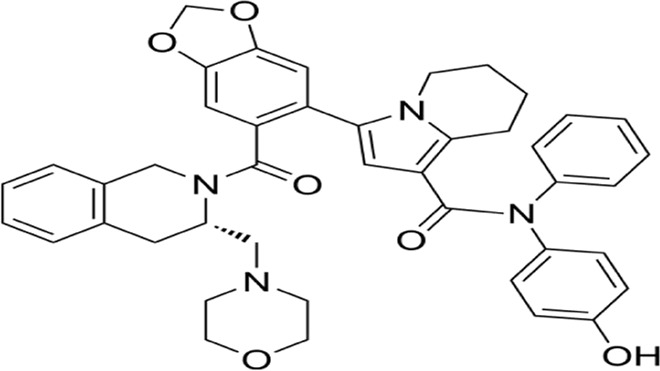	Direct BAX activator; bypasses anti-apoptotic inhibition	Mitochondrial Apoptosis	Casara et al. (2018)
Tapotoclax AMG176	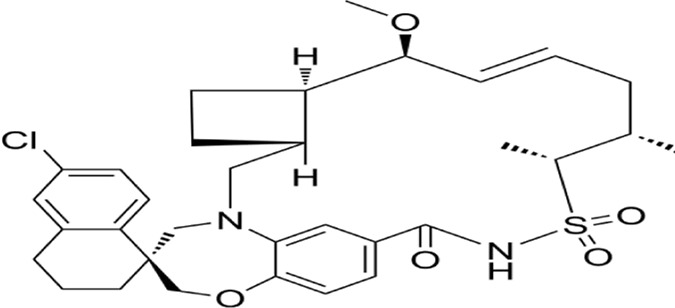	MCL-1 inhibitor; synergizes with BCL-2 inhibitors	Mitochondrial Apoptosis	Caenepeel et al. (2018)
Idelalisib	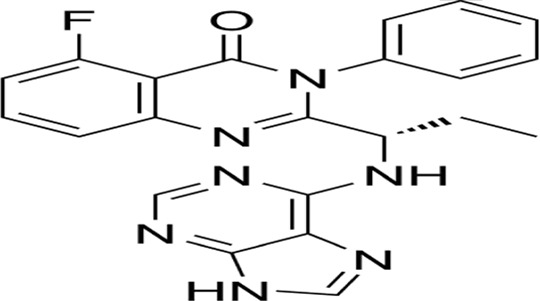	PI3K inhibitor; synergizes with BCL-2 inhibitors	Mitochondrial Apoptosis	Rahmani et al. (2018)
Doxorubicin	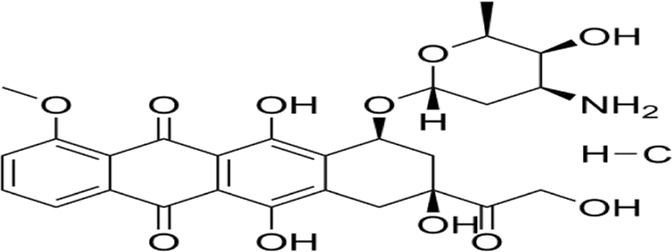	Induces pyroptosis via caspase-1/GSDMD cleavage	Pyroptosis	Fontana et al. (2024)
Berberine	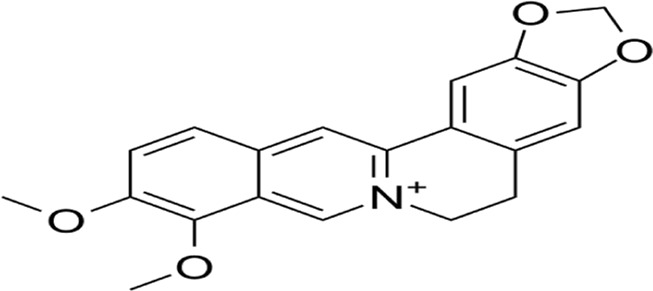	Pyroptosis inducer; activates multiple pathways	Pyroptosis	Wang et al. (2020)
Artemisinin Derivatives	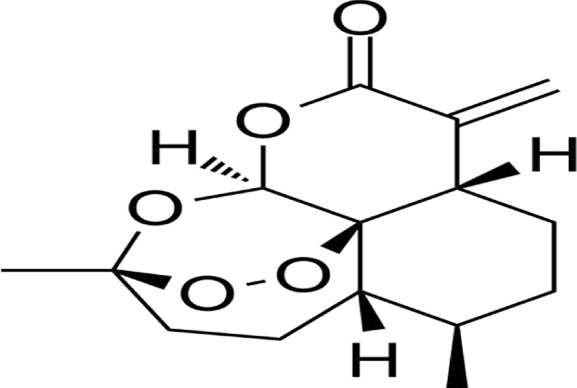	Pyroptosis inducer; high selectivity and low toxicity	Pyroptosis	Jiang et al. (2021b)
Ce6	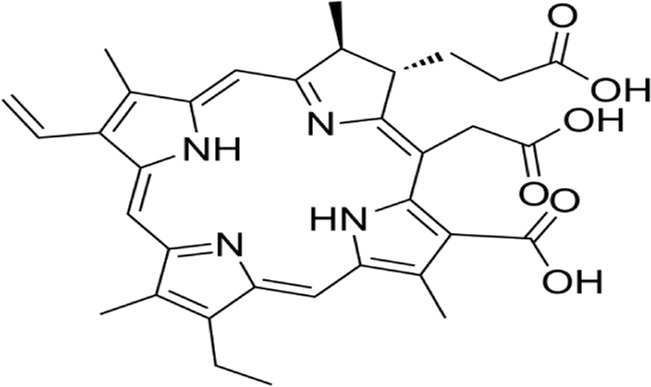	Photosensitizer; ROS generation synergizes with pyroptosis/necroptosis	Pyroptosis/Necroptosis	Li et al. (2024c)
Nivolumab	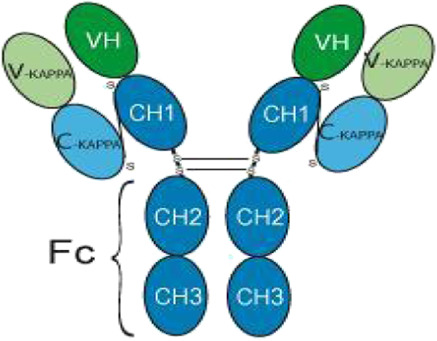	Anti-PD-1 monoclonal antibody; reverses T-cell exhaustion	Immune Checkpoint Blockade	Socinski et al. (2018)
Tisagenlecleucel	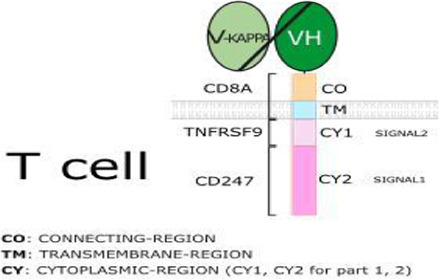	CAR-T therapy; targets CD19/BCMA	Cellular Immunotherapy	Davila et al. (2014)
